# Phenotypic and genetic associations between anhedonia and brain structure in UK Biobank

**DOI:** 10.1038/s41398-021-01522-4

**Published:** 2021-07-16

**Authors:** Xingxing Zhu, Joey Ward, Breda Cullen, Donald M. Lyall, Rona J. Strawbridge, Laura M. Lyall, Daniel J. Smith

**Affiliations:** 1 Institute of Health and Wellbeing, University of Glasgow, Glasgow, UK; 2 Cardiovascular Medicine Unit, Department of Medicine Solna, Karolinska Institutet, Stockholm, Sweden; 3 Health Data Research (HDR), Glasgow, UK; 4 Division of Psychiatry, Kennedy Tower, Royal Edinburgh Hospital, Edinburgh, UK

**Keywords:** Psychiatric disorders, Biomarkers

## Abstract

Anhedonia is a core symptom of multiple psychiatric disorders and has been associated with alterations in brain structure. Genome-wide association studies suggest that anhedonia is heritable, with a polygenic architecture, but few studies have explored the association between genetic loading for anhedonia—indexed by polygenic risk scores for anhedonia (PRS-anhedonia)—and structural brain imaging phenotypes. Here, we investigated how anhedonia and PRS-anhedonia were associated with brain structure within the UK Biobank cohort. Brain measures (including total grey/white matter volumes, subcortical volumes, cortical thickness (CT) and white matter integrity) were analysed using linear mixed models in relation to anhedonia and PRS-anhedonia in 19,592 participants (9225 males; mean age = 62.6 years, SD = 7.44). We found that state anhedonia was significantly associated with reduced total grey matter volume (GMV); increased total white matter volume (WMV); smaller volumes in thalamus and nucleus accumbens; reduced CT within the paracentral cortex, the opercular part of inferior frontal gyrus, precentral cortex, insula and rostral anterior cingulate cortex; and poorer integrity of many white matter tracts. PRS-anhedonia was associated with reduced total GMV; increased total WMV; reduced white matter integrity; and reduced CT within the parahippocampal cortex, superior temporal gyrus and insula. Overall, both state anhedonia and PRS-anhedonia were associated with individual differences in multiple brain structures, including within reward-related circuits. These associations may represent vulnerability markers for psychopathology relevant to a range of psychiatric disorders.

## Introduction

Anhedonia, defined as subjectively diminished capacity to experience pleasure, is a transdiagnostic symptom present in several psychiatric disorders, such as major depressive disorder (MDD) and schizophrenia [1, 2]. Anhedonia can be both a stable, trait-like characteristic and a transient state that fluctuates over time and with severity of psychiatric disorders, such as MDD [3]. In schizophrenia, trait anhedonia is associated with state anhedonia and may be independent of negative symptoms, but state anhedonia is correlated with both negative symptoms and depression [4]. In recent years, with the development of the Research Domain Criteria (RDoC) initiative [5], research efforts focused on transdiagnostic symptoms such as anhedonia have grown substantially, including progress on describing the underlying genetic architecture of anhedonia [6] and related neurobiological markers [7]. Anhedonia represents a promising RDoC construct with potential to elucidate some of the underlying biology of psychiatric disorders.

Anhedonia is mainly associated with impairments in reward processing and frontal-striatal brain circuits [8–10]. Evidence from neuroimaging studies suggests that alterations in reward-related regions may contribute to the subjective experience of anhedonia. Reductions in grey matter volume (GMV) and cortical thickness (CT) within several brain areas (including caudate, nucleus accumbens (NAcc), anterior cingulate cortex, prefrontal cortex and parietal lobe) have been reported in studies of trait anhedonia in patients with schizophrenia [11], in studies of negative symptoms of schizophrenia [12, 13], as well as in individuals with MDD [14]. Within non-clinical populations, trait anhedonia has been found to be associated with reduced volume in caudate [15] and NAcc [16]. Similarly, anhedonic depression was associated with reduced NAcc volume in a non-clinical sample [17]. In addition, reduced bilateral putamen volume was prospectively predictive for anhedonia severity while baseline anhedonia, depression and anxiety symptoms were controlled for [16]. However, null findings have also been reported. Yang et al. [18] found no correlation between state or trait anhedonia and prefrontal CT or parietal CT in healthy controls or subjects with MDD. This inconsistency may be due to relatively small sample sizes (generally less than 100 [11, 13–18]) and both demographic and clinical heterogeneity within samples. There is therefore a need for large population-based neuroimaging studies to better understand the neural correlates of anhedonia.

Anhedonia may also be associated with abnormalities of structural connectivity between brain regions, although findings have not been consistent. A study of negative symptoms in schizophrenia reported negative associations between anhedonia and fractional anisotropy (FA) of the left frontal lobe [19]. Another study found trait anhedonia was positively correlated with FA in the cingulum and superior longitudinal fasciculus in patients with schizophrenia [20]. In addition, Coloigner et al. [21] found that in depressed patients higher anhedonia was correlated with greater FA in the superior longitudinal fasciculus and lower FA in the cingulum, genu of corpus callosum and posterior thalamic radiation. Further, Yang et al. [22] found that, in healthy controls, trait anhedonia was associated with higher mean FA in the superior longitudinal fasciculus, anterior thalamic radiation and corticospinal tract. Despite the directional inconsistency, abnormalities in these structures tentatively support the assertion that anhedonia involves abnormal and/or inefficient communication between brain regions, especially structures within reward circuits, which might in turn contribute to vulnerability to psychiatric disorders, such as MDD [8, 9]. It is notable that these studies [20, 22] of white matter integrity were conducted with relatively small numbers of participants and, further, most studies on anhedonia and brain structure have been conducted in selected clinical samples of MDD or schizophrenia.

In contrast to recent large-scale genome-wide association studies (GWAS) of MDD and schizophrenia [23, 24], most GWAS of anhedonia have been small and underpowered. A meta-analysis of three GWAS (total sample size 6297) reported just a single locus associated with anhedonia in the discovery sample and no replication [25]. More recently, the largest GWAS (*n* = 375,275) of state anhedonia by our group identified 11 novel loci [6]. So far, to our knowledge, this study is also the first to have examined the association between PRS-anhedonia and brain structure [6]. In this paper, Ward et al. [6] examined associations between PRS-anhedonia and volume of 15 cortical/subcortical regions of interest and white matter integrity. Significant links were found with smaller total GMV, smaller volume of the orbitofrontal cortex, middle frontal gyrus, insula, and anterior temporal fusiform cortex, as well as worse white matter integrity.

In the present study, we extended the report by Ward et al. [6] by adding large-scale analyses for the state anhedonia phenotype and by including additional brain imaging parameters, particularly CT across the whole brain. We tested for associations between state anhedonia, PRS-anhedonia and brain structure (whole-surface CT, subcortical volumes and white matter integrity) and we examined the specificity of associations. Our hypothesis was that anhedonia would be associated with individual differences in brain structures, such as smaller NAcc, thinner anterior cingulate cortex [13, 16], and poor white matter integrity, and that PRS-anhedonia would also be associated with reward-related brain regions.

## Methods and materials

### Participants

The sample included in this study was drawn from UK Biobank, which gathered extensive questionnaire, physical and cognitive measures, as well as biological samples from over 500,000 participants. Informed consent was obtained from all participants, and this study was conducted under approval from the NHS National Research Ethics Service (UK Biobank approved applications #6553 and #17689).

At the first imaging visit, a total of 31,064 participants responded to the question about anhedonia (data field 2060). Participants were excluded for the following reasons: having a developmental or neurological disorder (see Table S1 for detailed participant exclusion criteria); not of White European ancestry; unclear values of anhedonia (responded as ‘prefer not to answer’ or ‘do not know’); age at magnetic resonance imaging (MRI) result missing; total intracranial volume (ICV, sum of total grey matter, white matter and ventricular cerebrospinal fluid volume) missing or beyond three standard deviations from the sample mean. A total of 19,592 participants (ages 45–80 years, 9225 males) were included in this study (see Table 1). For PRS-anhedonia analyses, participants included in the GWAS of anhedonia were also excluded to avoid overlap between the train and test samples.

**Table 1:** Descriptive statistics for demographic variables (*N* = 19,592).

Variable	*N* (%) or mean ± SD (range)
Gender	Male: 9225 (47.09%)
Age (years)	62.60 ± 7.44 (45–80)
Townsend deprivation index at recruitment (*N* = 19,577)	−2.06 ± 2.59 (−6.26–9.16)
Childhood traumatic events (*N* = 14,100)	1.70 ± 2.35 (0–20)
Adulthood traumatic events (*N* = 13,937)	1.97 ± 2.46 (0–20)
Frequency of unenthusiasm/disinterest in last 2 weeks	Not at all: 16,488 (84.16%)
	Several days: 2589 (13.21%)
	More than half the days:314 (1.60%)
	Nearly every day: 201 (1.03%)
Frequency of depressed mood in last 2 weeks (*N* = 19,257)	Not at all: 15,904 (82.59%)
	Several days: 2381 (14.7%)
	More than half the days:320 (1.66%)
	Nearly every day: 202 (1.05%)
Unprescribed/prescribed medication use	Yes: 3275(16.72%)
	No: 16,294 (83.17%)
	Prefer not to answer: 23 (0.12%)
Body mass index	Underweight: 125 (0.64%)
	Normal: 7580 (38.69%)
	Overweight: 7916 (40.40%)
	Obese: 3542 (18.08%)
	Missing: 429 (2.19%)
Current tobacco smoking	No: 18,853 (96.23%)
	Only occasionally: 442 (2.26%)
	Yes, on most or all days: 294 (1.50%)
	Prefer not to answer: 3 (0.02%)
Alcohol intake frequency	Daily or almost daily: 3283 (16.76%)
	Three or four times a week: 5644 (28.81%)
	Once or twice a week: 5312 (27.11%)
	One to three times a month: 2264 (11.56%)
	Special occasions only: 1969 (10.05%)
	Never: 1 118 (5.71%)
	Prefer not to answer: 2 (0.01%)
Education qualification	College or University degree: 8159 (46.65%)
	A levels/AS levels or equivalent: 2165 (12.41%)
	O levels/GCSEs or equivalent: 3342 (19.14%)
	CSEs or equivalent: 643 (3.84%)
	NVQ or HND or HNC or equivalent: 1089 (6.11%)
	Other professional qualifications eg: nursing: 882 (5.08%)
	Prefer not to answer: 52 (0.30%)
	None of the above: 1160 (6.47%)

### State anhedonia phenotype

As previously described [6], state anhedonia was assessed by a single question, *“Over the past two weeks, how often have you had little interest or pleasure in doing things?”*. Participants could choose from the following answers: “not at all”; “several days”; “more than half the days”; and “nearly every day”, which were coded as 0, 1, 2 and 3, respectively. This is identical to a question from the clinical interview for depression in the Diagnostic and Statistical Manual of Mental Disorders (DSM–5) and is a part of the depression scale in the Patient Health Questionnaire [26] and its short versions such as the 2-item Patient Health Questionnaire [27]. This demonstrates that it is a widely used and representative question in screening depression.

### Brain imaging variables

All neuroimaging data were acquired, pre-processed, quality controlled and made available by UK Biobank (https://biobank.ctsu.ox.ac.uk/crystal/crystal/docs/brain_mri.pdf). Details on the acquisition parameters (http://biobank.ctsu.ox.ac.uk/crystal/refer.cgi?id=1977) and the imaging protocol (http://biobank.ctsu.ox.ac.uk/crystal/refer.cgi?id=2367) are documented online and are described within protocol papers [28]. The neuroimaging data analysed in the current study consisted of: (1) total GMV and total white matter volume (WMV); (2) subcortical volumes; (3) CT of 31 regions in each hemisphere from the “Desikan–Killiany–Tourville” (DKT) protocol [29] over whole brain surface (Fig. S1); and (4) white matter microstructure, indexed by FA and mean diffusivity (MD). A more detailed description of these variables is provided within supplementary materials.

### Derivation of polygenic risk scores for anhedonia

The polygenic risk score for anhedonia was calculated using LDpred [30] based on the summary statistics from the GWAS of state anhedonia mentioned earlier [6]. Of note, the individuals with neuroimaging data in the current study were excluded from this GWAS [6]. Additional exclusion criteria for participants in the current study included: over 10% of genetic data missing; self-reported sex did not match genetic sex; purported sex chromosome aneuploidy was reported; where the heterozygosity value was a clear outlier; and participants were not of White European ancestry. In total, 16,696 participants were included in PRS-anhedonia analyses.

### Statistical analysis

Data analysis was conducted using Stata. All brain measures were rescaled into zero mean and unitary standard deviation. For each brain outcome, as noted above, data points beyond three standard deviations from the sample mean were iteratively excluded. False Discovery Rate (FDR) correction at *p* < 0.05 was applied across 40 volume/thickness measures and white matter integrity indexes separately using ‘p.adjust’ function in R [31, 32].

For associations between state anhedonia and brain structures, anhedonia was set as an independent predictor, and each neuroimaging measure was set as a dependent variable. For bilateral brain measures, anhedonia × hemisphere interactions were firstly examined in a repeated measures format to determine whether analysis of left and right homologous structures separately was required, with sex, age, age^2^, hemisphere, ICV and scanner positions on the *x*, *y* and *z* axes set as covariates. Where there was a significant anhedonia × hemisphere interaction, analyses on both lateralised structures were conducted additionally. In the main analysis, the model included sex, age, age^2^, total ICV, scanner positions on the *x*, *y* and *z* axes and hemisphere as fixed effects using repeated measure design. For whole-brain or single structures, a general linear model was applied without controlling for hemisphere. For the PRS-anhedonia analyses, PRS-anhedonia was set as a predictor and genotype array and the first ten genetic principal components were added, in addition to the above covariates, for all association tests. Moreover, considering participants were assessed at two different centers, (16,600 at the Cheadle imaging center, 2992 at the Newcastle imaging center), we repeated analyses for anhedonia, PRS-anhedonia and brain structure adjusting for assessment sites additionally, and examined differences between the two assessment sites.

Furthermore, we conducted several sensitivity analyses to assess the robustness of any observed associations with brain structures. Specifically, we examined (1) the association between anhedonia as a dichotomous variable and brain measures emerged from main analyses; (2) the relationship between anhedonia and brain structure after excluding participants with mental illness (17,489 healthy people); (3) whether associations found in main analyses remained significant when potential confounding factors including childhood traumatic events, adulthood traumatic events, medication use, depressed mood, Townsend social deprivation index, education qualifications, body mass index, current tobacco use and alcohol intake frequency were set as additional covariates, with anhedonia included as another covariate in analyses for PRS-anhedonia and (4) whether brain structures associated with PRS-anhedonia were specific compared with the polygenic risk score for MDD. Details and additional covariates are described in supplementary materials.

## Results

### Demographics

State anhedonia was significantly negatively correlated with age (Pearson’s *r* = −0.114, *p* < 0.001; Spearman’s rho = −0.117, *p* < 0.001). Independent *t*-test showed there was no significant sex difference for anhedonia (female = 1.201 ± 0.005, male = 1.188 ± 0.005, *t* = 1.878, *p* = 0.060; Mann–Whitney U Tests: *z* = 1.366, *p* = 0.172). PRS-anhedonia was significantly associated with state anhedonia (*β* = 0.031, F_(1,16647)_ = 65.28, R-squared = 0.004, *p* < 0.001) and PRS-MDD (Pearson’s *r* = 0.241, *p* < 0.001). Associations between anhedonia and potential confounding variables mentioned above are shown in supplemental results.

### Associations between state anhedonia, brain morphometric measures and white matter integrity

**Fig. 1:**
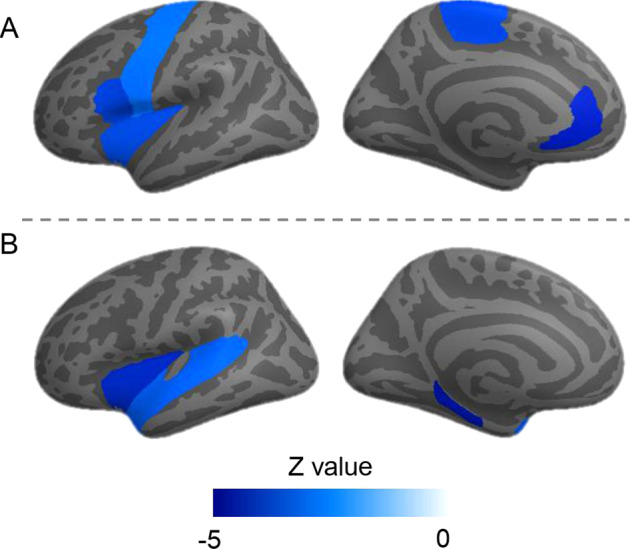
Cortical maps of associations between cortical thickness, state anhedonia and the polygenic risk score for anhedonia, rendered on the left hemisphere. **A** Regions associated with state anhedonia; **B** Regions associated with the polygenic risk score for anhedonia. Only regions survived multiple comparisons correction were shown. Negative *Z* values indicate cortical thinning in brain regions.

For whole-brain measures, we found that state anhedonia was associated with reduced total GMV (*β* = −0.025, *p*_corrected_ < 0.001; Table S2) and increased total WMV (*β* = 0.017, *p*_corrected_ < 0.001). For bilateral subcortical and cortical measures, no region demonstrated significant interaction with hemisphere, therefore no region was examined separately on different hemispheres (Table S3). Analyses using the repeated measure design showed there were significant associations between state anhedonia and smaller volume of the thalamus (*β* = −0.040, *p*_corrected_ < 0.001; Table S2) and NAcc (*β* = −0.051, *p*_corrected_ < 0.001), and with reduced CT in the paracentral gyrus (*β* = −0.040, *p*_corrected_ = 0.010), rostral anterior cingulate cortex (*β* = −0.042, *p*_corrected_ < 0.001; Fig. 1A), precentral (*β* = −0.033, *p*_corrected_ = 0.044), insula (*β* = −0.038, *p*_corrected_ = 0.010) and opercular part of inferior frontal gyrus (pars opercularis; *β* = −0.040, *p*_corrected_ = 0.007). Analyses controlling for assessment sites found same results (Table S4). Differences between the two assessment centers were showed in Table S5–S6.

**Fig. 2:**
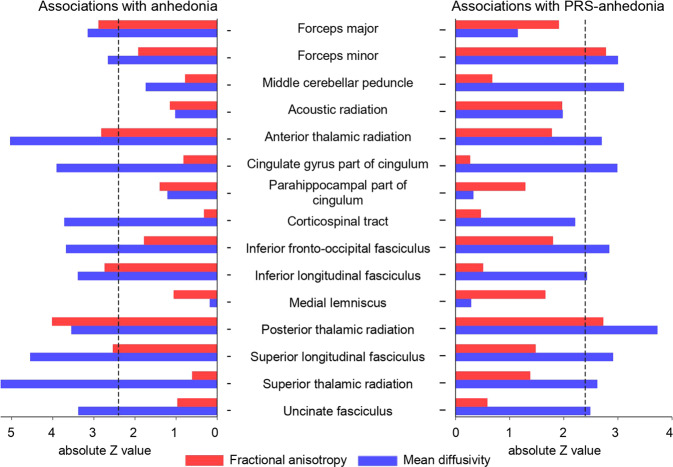
Associations between anhedonia (left panel) and polygenic risk for anhedonia (right panel) and white matter integrity. The *y*-axis represents the white matter tracts tested in our analyses. The *x*-axis represents the absolute *Z* values for anhedonia and polygenic risk for anhedonia in regression models. Fractional anisotropy and mean diffusivity are separately shown in red and blue. The absolute *Z* = 2.4 black dashed lines indicates significance. This value was chosen according to the significance of each regression model. In the left panel, bars to the left of the black dashed line indicate significant associations between anhedonia and corresponding white matter tracts. In the right panel, bars to the right of the black dashed line are significant, indicating associations between polygenic risk for anhedonia and white matter tracts.

The same models were applied to analyses of white matter tracts (FA and MD). We firstly tested the interaction of hemisphere and state anhedonia and found significant effects on MD of the acoustic radiation (*β* = −0.050, *p*_corrected_ = 0.012; Table S3) and MD of the uncinate fasciculus (*β* = 0.032, *p*_corrected_ = 0.012). Therefore, the left and right MD values of the two tracts were also tested (Table S2). We found anhedonia was associated with reduced FA in the forceps major (*β* = −0.043, *p*_corrected_ = 0.010; Table S2; Fig. 2), anterior thalamic radiation (*β* = −0.039, *p*_corrected_ = 0.012), inferior longitudinal fasciculus (*β* = −0.038, *p*_corrected_ = 0.014), posterior thalamic radiation (*β* = −0.055, *p*_corrected_ < 0.001) and superior longitudinal fasciculus (*β* = −0.036, *p*_corrected_ = 0.022). In addition, state anhedonia was associated with increased MD in the forceps major (*β* = 0.045, *p*_corrected_ = 0.006), forceps minor (*β* = 0.037, *p*_corrected_ = 0.017), left acoustic radiation (*β* = 0.035, *p*_corrected_ = 0.030), anterior thalamic radiation (*β* = 0.064, *p*_corrected_ < 0.001), cingulate gyrus part of cingulum (*β* = 0.052, *p*_corrected_ < 0.001), corticospinal tract (*β* = 0.051, *p*_corrected_ < 0.001), inferior fronto-occipital fasciculus (*β* = 0.049, *p*_corrected_ = 0.001), inferior longitudinal fasciculus (*β* = 0.045, *p*_corrected_ = 0.003), posterior thalamic radiation (*β* = 0.046, *p*_corrected_ < 0.001), superior longitudinal fasciculus (*β* = 0.064, *p*_corrected_ < 0.001), superior thalamic radiation (*β* = 0.066, *p*_corrected_ < 0.001), uncinate fasciculus (*β* = 0.042, *p*_corrected_ = 0.003), left uncinate fasciculus (*β* = 0.029, *p*_corrected_ = 0.045) and right uncinate fasciculus (*β* = 0.056, *p*_corrected_ < 0.001).

### PRS-anhedonia, brain morphometric measures and white matter integrity

Analyses of whole-brain measures found that PRS-anhedonia was associated with lower total GMV (*β* = −0.014, *p*_corrected_ < 0.001; Table S2) and higher total WMV (*β* = 0.012, *p*_corrected_ < 0.001). There was no significant interaction between PRS-anhedonia and hemisphere on subcortical volumes, as well as regional CT (Table S3). We found significantly negative associations between PRS-anhedonia and CT in the insula cortex (*β* = −0.030, *p*_corrected_ < 0.001; Table S2; Fig. 1B), parahippocampal cortex (*β* = −0.032, *p*_corrected_ < 0.001) and superior temporal gyrus (*β* = −0.024, *p*_corrected_ = 0.008). Same results were found when the assessment site was additionally adjusted for (Table S4). Differences between the two assessment centers were showed in Tables S5 and S7.

None of the measures of white matter integrity demonstrated significant interaction of hemisphere and PRS-anhedonia (Table S3). PRS-anhedonia was associated with lower FA in the forceps minor (*β* = −0.022, *p*_corrected_ = 0.021; Table S2; Fig. 2) and posterior thalamic radiation (*β* = −0.020, *p*_corrected_ = 0.023). In addition, analyses for MD values found positive associations with the forceps minor (*β* = 0.023, *p*_corrected_ = 0.021), middle cerebellar peduncle (*β* = 0.025, *p*_corrected_ = 0.021), anterior thalamic radiation (*β* = 0.019, *p*_corrected_ = 0.023), cingulate gyrus part of cingulum (*β* = 0.022, *p*_corrected_ = 0.021), inferior fronto-occipital fasciculus (*β* = 0.021, *p*_corrected_ = 0.021), inferior longitudinal fasciculus (*β* = 0.018, *p*_corrected_ = 0.038), posterior thalamic radiation (*β* = 0.027, *p*_corrected_ < 0.001), superior longitudinal fasciculus (*β* = 0.022, *p*_corrected_ = 0.021), superior thalamic radiation (*β* = 0.018, *p*_corrected_ = 0.027) and uncinate fasciculus (*β* = 0.017, *p*_corrected_ = 0.035).

### Sensitivity analyses: associations between state anhedonia (as a dichotomous variable) and brain structure

In general, analyses for anhedonia as a dichotomous variable found similar results (Table S8). However, compared to previous results, the paracentral gyrus (*β* = −0.035, *p*_uncorrected_ = 0.047) and insula (*β* = −0.034, *p*_uncorrected_ = 0.048) became only nominally significant before FDR correction while precentral cortex (*β* = −0.024, *p*_uncorrected_ = 0.167) was no longer significant. Associations with MD in the left acoustic radiation (*β* = 0.037, *p*_uncorrected_ = 0.068) and left uncinate fasciculus (*β* = 0.029, *p*_uncorrected_ = 0.104) were also no longer significant. See supplemental materials for more details.

### Sensitivity analyses: associations between state anhedonia (linear variable) and brain structure in healthy participants

For total GMV/WMV, subcortical volumes and regional CT, we found similar results. However, for white matter integrity, only FA in the posterior thalamic radiation (*β* = −0.045, *p*_uncorrected_ = 0.048; Table S9) and MD in the superior thalamic radiation remained significant (*β* = 0.043, *p*_uncorrected_ = 0.048). In addition, we found nominally significant interaction effect on several white matter tracts before FDR correction (Table S10; supplemental results). The Fig. S2 illustrates the interaction between anhedonia and mental health status on several white matter tracts. Briefly, similar patterns were found in participants with and without mental illness, but the association was stronger in those with mental illness.

### Sensitivity analyses: associations between state anhedonia, PRS-anhedonia and brain structure controlling for potential confounding factors

For state anhedonia, when additional covariates were added in the model, we found significant associations with increased total WMV (*β* = 0.018, *p*_corrected_ = 0.022; Table S11), smaller total GMV (*β* = −0.025, *p*_corrected_ = 0.009) and nucleus accumbens (*β* = −0.043, *p*_corrected_ = 0.038), as well as reduced thickness in the paracentral cortex (*β* = −0.062, *p*_corrected_ = 0.011), pars opercularis (*β* = −0.070, *p*_corrected_ = 0.009) and precentral cortex (*β* = −0.063, *p*_corrected_ = 0.011). For white matter integrity, none of those tracts survived FDR correction.

For PRS-anhedonia, we found PRS-anhedonia was still significantly associated with total GMW (*β* = −0.007, *p*_corrected_ = 0.020; Table S12), total WMV (*β* = 0.009, *p*_corrected_ = 0.005), as well as CT in the parahippocampal cortex (*β* = −0.031, *p*_corrected_ < 0.001), superior temporal cortex (*β* = −0.023, *p*_corrected_ = 0.011) and insula (*β* = −0.027, *p*_corrected_ = 0.005), while associations with FA/MD of white matter tracts were no longer significant.

### Associations between PRS-MDD and brain structure

Analyses of whole-brain measures and regional CT found nominally significant association with CT in the lateral occipital cortex (*β* = 0.017, *p*_uncorrected_ = 0.018; Table S13), posterior cingulate cortex (*β* = −0.016, *p*_uncorrected_ = 0.013) and insula (*β* = −0.015, *p*_uncorrected_ = 0.036), but none of them survived FDR correction. However, there were significant associations with lower FA or higher MD of several white matter tracts such as the anterior thalamic radiation and the cingulate gyrus part of cingulum. See supplemental results for further details.

## Discussion

Overall, we found that the phenotype of state anhedonia and the PRS-anhedonia were both associated with individual differences in brain structure. State anhedonia was associated with smaller volumes in the thalamus and NAcc, and with thinner CT in the paracentral cortex, the opercular part of inferior frontal gyrus, precentral cortex, insula and rostral anterior cingulate cortex, as well as poor integrity of many white matter tracts. Higher polygenic risk for anhedonia was associated with reduced total GMV, increased total WMV, thinner parahippocampal cortex, superior temporal cortex and insula cortex, plus poorer white matter integrity. Sensitivity analyses for anhedonia found consistent associations with total GMV, total WMV, NAcc and CT in pars opercularis. Similarly, associations between PRS-anhedonia and total GMV, total WMV and regional CT remined significant when potential confounding factors were considered.

### State anhedonia and brain structures

Consistent with previous studies, the main analyses for state anhedonia found associations with loss of grey matter in the thalamus, NAcc and rostral anterior cingulate cortex, consistent with the fronto-striatal reward circuit hypothesis [8, 33]. Previous studies have demonstrated associations with reduced volume or CT in these regions within non-clinical populations [16, 17], as well as in patients with schizophrenia and MDD [13, 34–36]. These brain structures are also key regions in the subcortical-cortical midline system, which has been related to abnormalities of the self in patients with MDD [37]. A recent study found that the NAcc was also associated with auditory hallucinations [38]. Furthermore, the negative associations between state anhedonia and CT in the paracentral cortex and pars opercularis are consistent with findings in patients with schizophrenia [12,13,39,40,], corresponding to the potential role of these areas in reward processing [41, 42].

Additionally, we found that state anhedonia was associated with poor white matter integrity, specifically lower FA and higher MD values within several white matter tracts. Unlike previous contradictory findings, the results for different tracts in this large sample were more consistent. Regarding specific white matter tracts, altered FA in the cingulum, superior longitudinal fasciculus, anterior thalamic radiation and corticospinal tract have been reported in relation to trait anhedonia [20, 22], in line with their function in reward processing [43]. Previous studies in patients with MDD and schizophrenia also reported worse white matter integrity of many tracts, such as the superior longitudinal fasciculus and anterior thalamic radiation observed in this study [44–46]. However, it is noteworthy that many anhedonia-related white matter tracts were no longer statistically significant when participants with mental illness were excluded. In addition, interaction analyses revealed stronger associations in those with mental illness, suggesting that the findings of white matter integrity might be driven by participants with mental illness. More studies are needed to elucidate the relationship between anhedonia and white matter in both healthy people and patients with mental illness.

### PRS-anhedonia and brain structures

Our analyses for PRS-anhedonia found associations with reduced total GMV and increased total WMV, along with reduced CT in the parahippocampal cortex, superior temporal gyrus and insula cortex. Previous findings on total GMV and total WMV in relation to PRS for schizophrenia/MDD are inconsistent. One study [47] reported reduced total brain volume and total WMV in relation to PRS for schizophrenia and no significant association with GMV, while later research found no associations with GMV or WMV [48–50]. However, it is notable that these studies had relatively small sample sizes (ranging from 152 to 1470) and may have lacked power to detect associations.

Regarding subcortical volume measures, we found no significant association with PRS-anhedonia, consistent with previous studies of PRS for schizophrenia and MDD [48, 50, 51]. Although anhedonia was found to be genetically correlated with NAcc volume [52], we failed to detect a significant association. For cortical regions, our prior work [6] on GMV also reported negative associations in the insular and temporal cortex, in addition to the findings on CT described in the current study. Importantly, in comparison to findings for PRS-MDD, associations with these brain areas were specific to PRS-anhedonia, indicating the potential contribution of genetic risk for anhedonia as a tool to reveal vulnerability biomarkers for mental illness. Previous studies of CT in patients with schizophrenia and MDD have also reported widespread thinning of the cortex, including parahippocampal cortex, superior temporal gyrus and insula cortex [40, 53]. Moreover, studies on polygenic risk for mental illness genetically related to anhedonia (PRS for schizophrenia) also point to links with reduced insular CT [54]. A meta-analysis for emotion/reward tasks in relation to anhedonia also observed associations with altered activation in widespread regions including parahippocampal cortex, superior temporal gyrus and insula cortex in healthy controls and in patients with schizophrenia or MDD [55]. These findings support the association between anhedonia and reward-related processes.

PRS-anhedonia was also associated with poor white matter integrity. We found lower FA and higher MD of several white matter tracts in relation to PRS-anhedonia, such as the superior longitudinal fasciculus, the cingulate gyrus part of cingulum, and the posterior thalamic radiation. This is in line with the previously reported results by Ward et al. [6]. Given that there are potential shared genetic components between white matter integrity and MDD and schizophrenia [56–58]—and genetic correlations between anhedonia, MDD and schizophrenia [6, 25]—it is perhaps not surprising that we observed associations between polygenic risk for anhedonia and white matter indices. In over 9000 participants from UK Biobank, Shen et al. [59] recently found PRS for MDD was associated with white matter integrity of several tracts, such as the superior longitudinal fasciculus, the cingulate gyrus part of cingulum, the inferior fronto-occipital fasciculus and superior thalamic radiation, which were associated with PRS-anhedonia in our study. Previous studies on PRS for MDD have also reported associations with decreased white matter integrity, most notably in the superior longitudinal fasciculus, cingulum and thalamic radiations [60–62], although null findings have also been reported with respect to polygenic risk for MDD or schizophrenia [48, 63]. Together with our findings of worse white matter integrity in relation to state anhedonia, white matter tracts associated with PRS-anhedonia could represent a biomarker of vulnerability to anhedonia and psychiatric disorders.

We found an overlap of brain structural correlates between state anhedonia and PRS-anhedonia. The results of morphometric measures of brain regions after FDR correction indicated heterogeneity of related regions, with only a weak overlap (insula CT) identified, and it became non-significant when potential confounding factors were considered. For white matter integrity, we found common associations with many white matter tracts such as the forceps minor, superior longitudinal fasciculus, anterior thalamic radiation and posterior thalamic radiation, although they disappeared when those additional covariates were added in the model. Combining the main results and sensitivity analyses, total GMV and total WMV showed the strongest association with both state anhedonia and PRS-anhedonia. Considering previous studies reporting mediating roles of brain measures in the relationship between genetic risk and psychiatric symptoms [59, 64], and the overlapping strong associations found in this study, we therefore also conducted mediation analyses to explore whether the two brain measures or state anhedonia were potential mediation candidates of genetic risk for anhedonia. Details of the statistics and results are provided within supplementary materials. Briefly, our findings support that total GMV, total WMV and state anhedonia may mediate the influence of genetic loading for anhedonia (Fig. S3). Future studies are needed to clarify the potential mechanisms linking brain alterations, state anhedonia and genetic risk for anhedonia.

### Integrating findings of morphometric measures and white matter integrity

This study performed multimodal structural brain imaging analyses on subcortical volume, regional CT and white matter integrity, which enabled us to carry out more comprehensive analyses. Although the morphometric measures and white matter indices are distinct and were analysed in parallel, the results together suggest a role for reward circuits.

The thalamus, NAcc and rostral anterior cingulate cortex lie within the reward circuit. White matter tracts such as the cingulum bundle and the anterior thalamic radiation are also involved in reward processing. Specifically, the cingulum is the most prominent tract in the limbic system, directly connecting several reward-related regions, such as the anterior cingulate cortex, orbital frontal cortex, hippocampus, parahippocampal region and thalamic nucleus, and has been previously reported to be involved in executive cognition and emotion processing [43]. In addition, the anterior thalamic radiation connects the thalamus, prefrontal cortex, and ventral periaqueductal grey, with fibres extending to temporal medial regions (e.g., amygdala and hippocampus) [65]. Its horizontal fibres are connected to areas that are also relevant in reward processing, such as the rostral anterior cingulate gyrus. Moreover, white matter integrity in the cingulate, anterior thalamic radiation and inferior fronto-occipital fasciculus, amongst others, has been found to be positively correlated with reward-related activity in NAcc [66]. Overall, morphometric alterations correspond to poor integrity of reward-related white matter tracts, supporting the reward circuit hypothesis of anhedonia [8, 33].

### Associations independent of potential confounding factors

Some external factors, such as childhood trauma and antipsychotic medication, may contribute to brain structural alterations [67, 68]. However, the influence of potential confounding factors associated with brain abnormalities in psychiatric disorders has often been neglected. Most previous studies on PRS for MDD and schizophrenia did not consider those factors [54, 69, 70], and this was the case in prior PRS-anhedonia analyses [6]. Our sensitivity analyses controlled for childhood traumatic events, adulthood traumatic events, medication use, depressed mood, Townsend social deprivation index, education qualification, body mass index, current tobacco use and alcohol intake frequency. The observed associations between anhedonia and total GMV/WMV, NAcc and CT in pars opercularis, and the associations between PRS-anhedonia and total GMV/WMV, CT within the parahippocampal cortex, superior temporal gyrus and insula were independent of these variables.

It should also be noted that the associations between anhedonia, PRS-anhedonia and white matter tracts were not statistically significant when these covariates were considered. Previous studies have demonstrated associations between factors such as childhood trauma, medication use, and white matter integrity [71, 72]. Future studies focusing on biomarkers of psychiatry disorders should consider the effects of these potential confounding factors and gene–environment interactions.

### Strengths and limitations

To our knowledge, this is the largest study to test for associations between anhedonia and brain parameters within a single population-based sample, and the first study to examine associations between anhedonia, PRS-anhedonia, and regional CT of the whole brain surface. Our supplementary analyses considered other covariates such as childhood traumatic events, medication use, depressed mood, tobacco use and alcohol intake frequency, highlighting robust associations driven by genetic risk (PRS-anhedonia).

Despite these strengths, there are some important potential limitations. Firstly, we note that participants in this study were aged from 45 to 80, which means that cumulative environmental risk may have contributed to some associations. Secondly, we only included participants of White European descent. Although the genetic basis of psychiatry disorders might be partially shared between different populations [73, 74], we should be cautious to generalize these results to other populations. Thirdly, considering that anhedonia may also manifest in childhood, before the onset of psychiatric disorders [7, 75], further consideration of how polygenic risk for anhedonia may influence child or adolescent brain development and brain changes over longer developmental periods will be important to further understand the nature of the relationship between anhedonia and brain structure. Moreover, the measurement of anhedonia was a single self-report item. A dimensional measure of anhedonia, such as the Chapman Physical and Social Anhedonia Scale [3] might prove to be a more informative phenotype in future work. Finally, the effect of the PRS-anhedonia is very modest; however, this is in line with those observed for other complex mental health traits [76, 77].

## Conclusion

In summary, we found that the phenotype of anhedonia and genetic risk for anhedonia were both associated with brain structures within a large population-based sample of 19,592 people. This included reduced volume/CT of total grey/white matter, thalamus, NAcc, paracentral gyrus, pars opercularis, rostral anterior cingulate cortex, parahippocampal cortex, superior temporal gyrus and insula, as well as worse white matter integrity. Overall, our findings suggest that reward-related brain structures are associated with anhedonia and its genetic risk and highlight potential neuroanatomical markers of risk for psychopathology across a range of psychiatric disorders.

## Supplementary information

supplement materials
