# Supplementary material for: Phenotypic and genetic associations between anhedonia and brain structure in UK Biobank
Source: Transl Psychiatry. 2021 Jul 16;11:395. doi: 10.1038/s41398-021-01522-4 (PMC8289859; doi:10.1038/s41398-021-01522-4)
Supplement: Supplementary file 1 — supplement materials [file 41398_2021_1522_MOESM1_ESM.docx]

# Phenotypic and genetic associations between anhedonia and brain structure in UK Biobank

# Supplementary materials

**Table of Contents**

Supplementary Methods

Supplementary Results

Figures S1-S3

Tables S1-S13

Supplementary References

# **Supplementary methods**

## MRI preprocessing

The T1-weighted volumes were pre-processed and analysed with the FMRIB Software Library (FSL) (<http://www.fmrib.ox.ac.uk/fsl>). Following removal of face area, gradient distortion correction, the brain was non-linearly warped to the MNI152 "nonlinear 6th generation" standard-space T1-weighted volume template, and the brain area of the images was then extracted for segmentation. Firstly, a tissue-type segmentation using FAST (FMRIB's Automated Segmentation Tool) was applied to extract cerebrospinal fluid, grey matter and white matter; then subcortical structures are extracted using FIRST (FMRIB's Integrated Registration and Segmentation Tool) and the volumes of thalamus, putamen, pallidum, hippocampus, caudate, amygdala and accumbens were calculated for further analysis (<https://biobank.ctsu.ox.ac.uk/crystal/crystal/docs/brain_mri.pdf>).

Furthermore, the T1 images were also processed with FreeSurfer (http://surfer.nmr.mgh.harvard.edu/). The technical details are described in previous publications ^1,2^. Briefly, the processing stream includes motion correction and averaging, removal of non-brain tissue, automated Talairach transformation, intensity normalisation, white matter segmentation, cortical surface reconstruction and parcellation. Cortical thickness was computed in FreeSurfer by calculating the closest distance from the gray/white matter boundary and the gray/CSF boundary at each vertex on the tessellated surface. Researchers compared four parcellation protocols implemented in FreeSurfer and recommended using Desikan-Killiany-Tourville (DKT) protocol approach to derive grey matter thickness ^3^. Therefore, this study adopted cortical thickness calculated according to the DKT atlas, with each hemisphere being parcellated into 31 regions (Figure S1).

Diffusion-weighted imaging data was initially corrected for eddy currents, head motion and outlier-slices, and then was processed by UK biobank with a probabilistic tractography based method using the BEDPOSTx tool (Bayesian Estimation of Diffusion Parameters Obtained using Sampling Techniques) and PROBTRACKx ^4^. Maps for fractional anisotropy (FA) and mean diffusivity (MD) were generated and FA maps were warped to standard space and used to generate the fibres. Twenty-seven tracts were generated by utilizing the standard-space start/stop ROI masks defined by AutoPtx ^5^, including forceps major, forceps minor and uncinate fasciculus and 12 bilateral tracts in both hemispheres.

## Measures of covariates

Genetic principal components 1–10, calculated via principal component analysis, were included as population stratification covariates.

Head positions in the MRI scanner were included as covariates, including lateral (<https://biobank.ctsu.ox.ac.uk/showcase/field.cgi?id=25756>), transverse (<http://biobank.ctsu.ox.ac.uk/crystal/field.cgi?id=25757>) and longitudinal (<https://biobank.ctsu.ox.ac.uk/showcase/field.cgi?id=25758>) co-ordinates of the centre of the brain mask within the scanner. Intracranial volume consists of grey matter, white matter and ventricular cerebral spinal fluid.

The score of childhood traumatic events was calculated via summing five online follow-up questions (<https://biobank.ndph.ox.ac.uk/showcase/label.cgi?id=145>): (1) "When I was growing up... I felt loved"; (2) "When I was growing up... People in my family hit me so hard that it left me with bruises or marks"; (3) "When I was growing up... I felt that someone in my family hated me"; (4) "When I was growing up... Someone molested me (sexually)"; (5) "When I was growing up... There was someone to take me to the doctor if I needed it". Participants answered to these questions by selecting “Prefer not to answer”, “Never true”, “Rarely true”, “Sometimes true”, “Often” or “Very often true”. The scores for “feeling loved” and “being taken to doctor when needed” are in an opposite direction, so they were reversed when calculating sum scores.

The score of adulthood traumatic events was also calculated via summing five online follow-up questions (<http://biobank.ndph.ox.ac.uk/showcase/label.cgi?id=145>): (1) "Since I was sixteen... I have been in a confiding relationship "; (2) "Since I was sixteen... A partner or ex-partner deliberately hit me or used violence in any other way "; (3) "Since I was sixteen... A partner or ex-partner repeatedly belittled me to the extent that I felt worthless "; (4) "Since I was sixteen... A partner or ex-partner sexually interfered with me, or forced me to have sex against my wishes "; (5) "Since I was sixteen... There was money to pay the rent or mortgage when I needed it ". Participants answered to these questions by selecting “Prefer not to answer”, “Never true”, “Rarely true”, “Sometimes true”, “Often” or “Very often true”. The scores for “in a confiding relationship” and “Able to pay rent/mortgage” are in an opposite direction, so they were reversed when calculating sum scores.

Considering only a minority of the sample used medication, we chose to examine impact of ever/never use of medication rather than the specific classes of psychotropic medication. In online follow-up assessments, participants who answered “Yes” to "Have you ever had a time in your life when you felt sad, blue, or depressed for two weeks or more in a row?" and "Have you ever had a time in your life lasting two weeks or more when you lost interest in most things like hobbies, work, or activities that usually give you pleasure?" reported substances taken for depression. Participants chose from “Unprescribed medication (more than once)”, “Medication prescribed to you (for at least two weeks)” or “Drugs or alcohol (more than once)” (<https://biobank.ndph.ox.ac.uk/showcase/field.cgi?id=20546>). We grouped participants according to use of medication. Those used unprescribed or prescribed medication were classified into one group, while the rest and healthy participants were included in another group.

Depressed mood was assessed by a single question, *"* *Over the past two weeks, how often have you felt down, depressed or hopeless?".* Participants could choose from the following answers: “not at all”; “several days”; “more than half the days”; and “nearly every day”, which were coded as 0, 1, 2 and 3 respectively.

Townsend deprivation index (<http://biobank.ndph.ox.ac.uk/showcase/field.cgi?id=189>) at recruitment is a score assigned based on census output regarding their postcode. Education qualification (<http://biobank.ndph.ox.ac.uk/showcase/field.cgi?id=6138>), current tobacco smoking (<http://biobank.ndph.ox.ac.uk/showcase/field.cgi?id=1239>) and alcohol intake frequency (<http://biobank.ndph.ox.ac.uk/showcase/field.cgi?id=1558>) at the first imaging visit were collected using touchscreen questions.

The body mass index (BMI; <http://biobank.ndph.ox.ac.uk/showcase/field.cgi?id=21001>) at the first imaging visit is calculated using a person's height and weight. The formula is BMI = kg/m^2^. Ordinal values of BMI were divided into four categories: underweight (BMI<18.5), normal (18.5≤BMI<25), overweight (25≤BMI<30) and obese (BMI>30).

Medication use, education qualification, current tobacco smoking and alcohol intake frequency and BMI were added to models as categorical variables.

## Sensitivity analyses

Analyses regarding anhedonia as a dichotomous variable were performed using same models. We coded anhedonia into ‘0’ and ‘1’, with value ‘0’ referring to participants who reported ‘not at all’ (N = 16488), and a value of ‘1’ representing the remainder of participants (who had reported any anhedonia, N = 3104) and re-analysed its relationship with brain measures from main analyses.

The association between anhedonia and brain structure in healthy participants was also analysed using the same models. To achieve a sample without people with mental illness, we excluded those who self-reported having depression, post-natal depression, schizophrenia, mania/bipolar disorder/manic depression, anxiety/panic attacks, post-traumatic stress disorder, anorexia/bulimia/other eating disorder or obsessive-compulsive disorder. In addition, the interaction between anhedonia and mental illness status (17,489 healthy people vs. 2103 reported any of above disorders) was examined with the same models except adding the mental illness status and an interaction term.

The polygenic risk score for major depressive disorder (PRS-MDD) were calculated using LDpred ^6^ applied to the summary statistics from a GWAS of major depression ^7^. This GWAS contained participants from UK Biobank so we used summary statistics that excluded both UK Biobank and 23andme. We adopted the same quality control criterion used in calculation of PRS-anhedonia and also standardized the scores and excluded values beyond three standard deviations from the sample mean.

## Mediation analysis

Previous studies on polygenic risk scores for major depressive disorder or schizophrenia have indicated that brain measures may serve as mediators between genetic risk and psychiatric symptoms. For example, Shen et al. ^8^ found that MD in anterior thalamic radiation mediated the effect of depression-PRS on current depressive symptoms and that current depressive symptoms mediated the effect of depression-PRS on global MD, MD in thalamic radiations and superior longitudinal fasciculus. Alloza et al. ^9^ also found the cortical thickness of the insula may mediate the relationship between PRS for schizophrenia and auditory hallucinations. By contrast, no study to date has examined mediation candidates of polygenic risk for anhedonia. Therefore, we conducted the mediation analyses in order to explore possible mediation candidates of PRS-anhedonia and to clarify the relationship between anhedonia, PRS-anhedonia and related brain measures.

Because we have not determined the directional or causal relationship of these associations, we tested whether brain structures might act as mediators between PRS-anhedonia and state anhedonia and whether state anhedonia could mediate the effect of PRS-anhedonia on brain structures. The brain measures were restricted to total grey matter volume (GMV) and total white matter volume (WMV), because only the two were significantly associated with both state anhedonia and PRS-anhedonia after controlling for all covariates. Sex, age, age^2^, ICV and scanner positions on the x, y and z axes, genotype array and the first ten genetic principal components were added in the model as covariates. Analyses were conducted using the ‘sem’ command in Stata and the significance of the mediation effect was estimated using the bootstrap approach (with 1000 random samplings).

# **Supplementary Results**

## Demographics regarding confounding covariates

There were significantly positive associations between anhedonia and depressed mood (Pearson's r = 0.662, p < 0.001; Spearman's rho = 0.627, p < 0.001), Townsend social deprivation index (Pearson's r = 0.161, p < 0.001; Spearman's rho = 0.073, p < 0.001), childhood traumatic events (Pearson's r = 0.172, p < 0.001; Spearman's rho = 0.140, p < 0.001) and adulthood traumatic events (Pearson's r = 0.078, p < 0.001; Spearman's rho = 0.157, p < 0.001). There was a significant difference in anhedonia between never/ever medication use groups (two sample t test: ever use group = 1.338 ± 0.011, never use group = 1.166 ± 0.004, t = -18.010, p < 0.001; Using Mann-Whitney U Tests: z = -19.318, p < 0.001). In addition, we found significant differences in anhedonia between different groups for BMI (One-way ANOVA: F = 60.28, p < 0.001; Kruskal-Wallis H test: χ2 = 64.25, *p* < 0.001), current tobacco smoking status (F = 53.32, p < 0.001; Kruskal-Wallis H test: χ2 = 38.69, *p* < 0.001), alcohol intake frequency (F = 21.90, p < 0.001; Kruskal-Wallis H test: χ2 = 40.53, *p* < 0.001), and education qualification (F = 18.18, p < 0.001; Kruskal-Wallis H test: χ2 = 34.49, *p* < 0.001). PRS-MDD was also significantly associated with state anhedonia (β = 0.025, F_(1,17293)_ = 44.37, R-squared = 0.003, p < 0.001). Participants with mental illness (1650 with depression and 453 with other mental disorders) reported higher state anhedonia than healthy individuals (healthy group = 1.162 ± 0.003, group with mental illness = 1.467 ± 0.016, t = -26.9, p < 0.001) and the Mann-Whitney U Test observed same difference (z = -26.344, p < 0.001).

## Influence of assessment centers on the associations between state anhedonia, PRS-anhedonia and brain structure

As the Table S4 showed, when the assessment center was included as an additional covariate, we found same brain alterations. As for the differences between the two sites, we firstly examined the interaction between anhedonia/PRS-anhedonia and assessment center on brain structures. Results showed no significant interaction between PRS-anhedonia and assessment center, but significant interaction between anhedonia and assessment center on mean diffusivity in the forceps minor, inferior fronto-occipital fasciculus, inferior longitudinal fasciculus and superior longitudinal fasciculus (Table S5). In addition, we further examined the associations between anhedonia/ PRS-anhedonia and brain structures that we observed in the main analyses (including above four white matter tracts) in the two assessment centers respectively. Results were shown in Table S6-S7. Briefly, for the Cheadle imaging center, we observed similar associations as we found in the main analyses. For the Newcastle center, associations were very weak because of the much smaller sample size. Moreover, looking closely at above four white matter tracts, the associations in two assessment centers seemed to be in different directions, but they were not significant in the Newcastle center.

## Associations between state anhedonia (as a dichotomous variable) and brain structure

State anhedonia was associated with reduced total GMV (β = -0.033, p_corrected_ <0.001; Table S8) and increased total WMV (β = 0.020, p_corrected_ = 0.002), smaller volume of the thalamus (β = -0.056, p_corrected_ < 0.001) and nucleus accumbens (β = -0.069, p_corrected_ < 0.001), and with reduced CT in the rostral anterior cingulate cortex (β = -0.043, p_corrected_ = 0.012) and the opercular part of inferior frontal gyrus (β = -0.048, p_uncorrected_ = 0.009). The paracentral gyrus (β = -0.035, p_uncorrected_ = 0.047) and insula (β = -0.034, p_uncorrected_ = 0.048) was nominally significant before FDR correction while precentral cortex (β = -0.024, p_uncorrected_ = 0.167) was not significant.

With regard to white matter integrity, state anhedonia remained significantly associated with reduced FA in the forceps major (β = -0.045, p_corrected_ = 0.029; Table S4), the anterior thalamic radiation (β = -0.043, p_corrected_ = 0.025), inferior longitudinal fasciculus (β = -0.046, p_corrected_ = 0.022) and posterior thalamic radiation (β = -0.074, p_corrected_ < 0.001) and superior longitudinal fasciculus (β = -0.049, p_corrected_ = 0.017). In addition, we found associations with increased MD in the forceps major (β = 0.049, p_corrected_ = 0.019), forceps minor (β = 0.047, p_corrected_ = 0.020), anterior thalamic radiation (β = 0.080, p_corrected_ < 0.001), cingulate gyrus part of cingulum (β = 0.054, p_corrected_ = 0.006), corticospinal tract (β = 0.057, p_corrected_ = 0.005), inferior fronto-occipital fasciculus (β = 0.059, p_corrected_ = 0.003), inferior longitudinal fasciculus (β = 0.053, p_corrected_ = 0.008), posterior thalamic radiation (β = 0.059, p_corrected_ = 0.003), superior longitudinal fasciculus (β = 0.085, p_corrected_ < 0.001), superior thalamic radiation (β = 0.088, p_corrected_ < 0.001), uncinate fasciculus (β = 0.046, p_corrected_ = 0.012) and right uncinate fasciculus (β = 0.063, p_corrected_ = 0.003). Associations with MD in the left acoustic radiation (β = 0.037, p_uncorrected_ = 0.068) and left uncinate fasciculus (β = 0.029, p_uncorrected_ = 0.104) were not significant.

## Associations between state anhedonia (linear variable) and brain structure in healthy participants

For total GMV/WMV, subcortical volumes and regional cortical thickness, we found similar results. State anhedonia was associated with reduced total GMV (β = -0.018, p_corrected_ = 0.002; Table S9) and increased total WMV (β = 0.011, p_corrected_ = 0.034), smaller volume of the thalamus (β = -0.041, p_corrected_ < 0.001) and NAcc (β = -0.057, p_corrected_ < 0.001), and with reduced CT in the paracentral gyrus (β = -0.044, p_corrected_ = 0.005), rostral anterior cingulate cortex (β = -0.045, p_corrected_ = 0.002), opercular part of inferior frontal gyrus (pars opercularis; β = -0.040, p_corrected_ = 0.009), insula (β = -0.033, p_corrected_ = 0.033) and precentral cortex (β = -0.032, p_corrected_ = 0.035).

However, for white matter integrity, only FA in the posterior thalamic radiation (β = -0.045, p_uncorrected_ = 0.048; Table S5) and MD in the Superior thalamic radiation remained significant (β = 0.043, p_uncorrected_ = 0.048). In addition, before FDR correction, nominally significant associations were observed with FA in the forceps major (β = -0.036, p_uncorrected_ = 0.038), as well as MD in the corticospinal tract (β = 0.039, p_uncorrected_ = 0.015), superior longitudinal fasciculus (β = 0.037, p_uncorrected_ = 0.023), anterior thalamic radiation (β = 0.034, p_uncorrected_ = 0.024) and posterior thalamic radiation (β = 0.033, p_uncorrected_ = 0.028). It seems that the association between anhedonia and white matter integrity is weaker when participants with mental disorders were excluded, compared with that of healthy participants (Table 2). Therefore, we subsequently examined the interaction of anhedonia and mental illness status (with/without mental illness) on white matter integrity.

We found nominally significant interaction effects on several white matter tracts before FDR correction (Table S10). Figure S2. illustrates the interaction between anhedonia and mental health status on several white matter tracts, including a) FA in the superior longitudinal fasciculus, b) MD in the cingulate gyrus part of cingulum, c) MD in the inferior longitudinal fasciculus, and d) MD in the uncinate fasciculus.

## Associations between PRS-MDD and brain structure

No significant interactions of PRS-MDD and hemisphere were observed for any brain structure. Analyses for white matter integrity found PRS-MDD was associated with lower FA in the posterior thalamic radiation (β = -0.019, p_corrected_= 0.036; Table S13), and higher MD in the forceps minor (β = 0.023, p_corrected_= 0.013), anterior thalamic radiation (β = 0.018, p_corrected_= 0.036), cingulate gyrus part of cingulum (β = 0.023, p_corrected_= 0.012), corticospinal tract (β = 0.021, p_corrected_= 0.023), inferior fronto-occipital fasciculus (β = 0.022, p_corrected_= 0.012), inferior longitudinal fasciculus (β = 0.022, p_corrected_= 0.013), posterior thalamic radiation (β = 0.023, p_corrected_= 0.010), superior longitudinal fasciculus (β = 0.027, p_corrected_< 0.001) and superior thalamic radiation (β = 0.024, p_corrected_= 0.010).

## Mediating candidates of PRS-anhedonia

 In the models examining the mediation effect of brain measures, we found that total GMV and total WMV significantly mediated the relationship between PRS-anhedonia and state anhedonia. Coefficients for individual paths are provided in Figure S3a. Total GMV and total WMV may mediate 2.7% and 2.2% of the impact of PRS-anhedonia on state anhedonia respectively. In addition, in the models with state anhedonia as mediators, we found state anhedonia may explain 4.9% of the association between PRS-anhedonia and total GMV, and 4.3% of the association between PRS-anhedonia and total WMV. Coefficients for individual paths are provided in Figure S3b.

Although the mediation effect is very small, it supports the potential mediating role of the brain in the relationship between genetic risk and state anhedonia and the possibility of anhedonia as a mediation candidate of genetic risk for anhedonia. This indicates that the relationship between psychosis and brain phenotypes may not be one-way causality and there may be complex interactions between psychosis and the brain. More studies are needed to clarify the associations between psychiatric disorders and the brain. We will also explore the directional or causal relationship between anhedonia and brain measures and identify putative neural mediators in the future.

# Supplementary figures

**Figure S1. Regions in the “Desikan-Killiany-Tourville” cortical labeling protocol ^10^.**


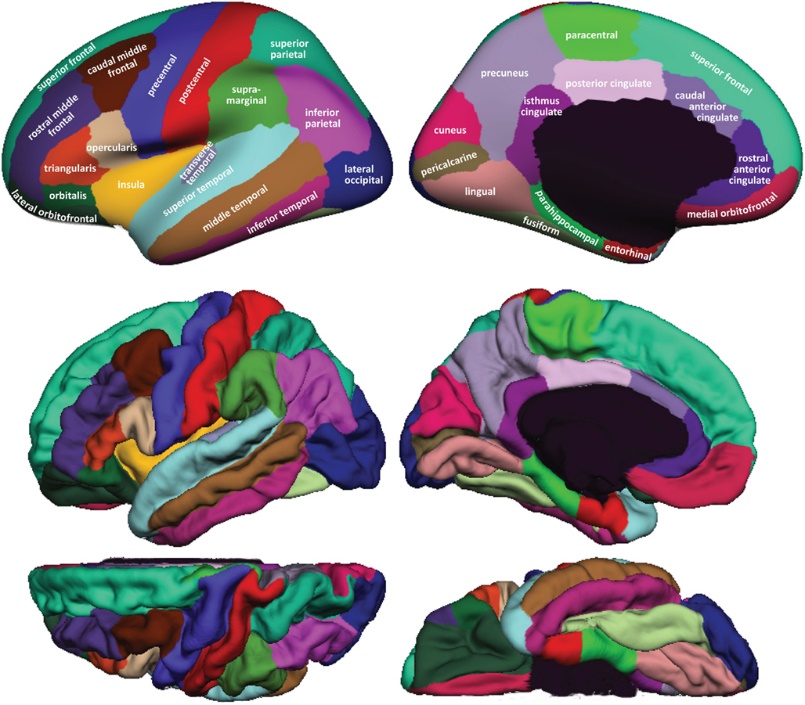


**Figure S2. The interaction between anhedonia and mental health status on several white matter tracts, including a) FA in the superior longitudinal fasciculus (SLF), b) MD in the cingulate gyrus part of cingulum (CC), c) MD in the inferior longitudinal fasciculus (ILF), and d) MD in the uncinate fasciculus (UF).** These plots suggest that the association between anhedonia and white matter integrity is stronger in participants with mental illness compared with that in those without mental illness. FA, fractional anisotropy; MD, mean diffusivity.


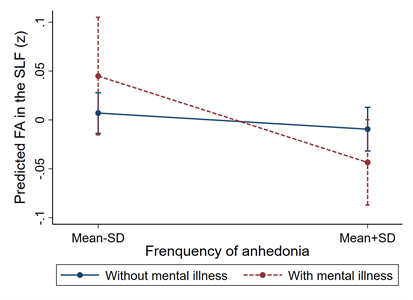

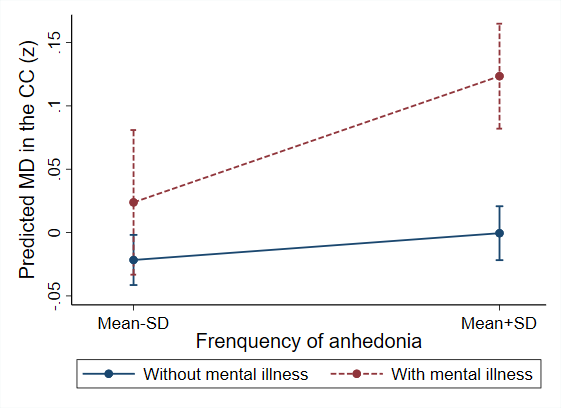
a) b)


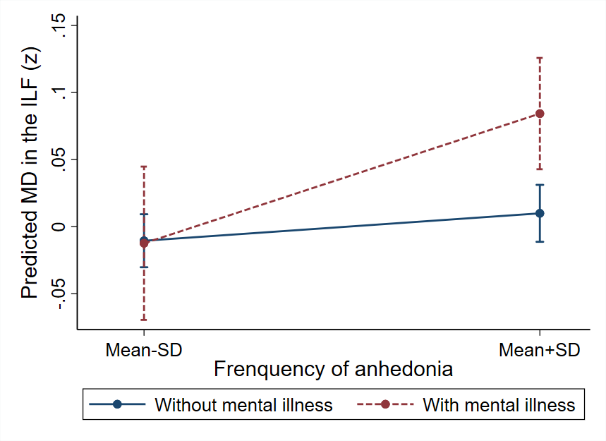

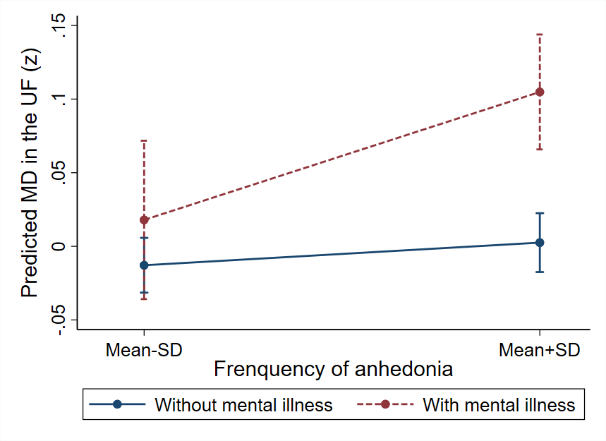
c) d)

**Figure S3. Path diagram of the mediation models of PRS for anhedonia, total grey matter volume, total white matter volume and state anhedonia.** a) Total grey/white matter volume partially mediates the effect of PRS for anhedonia on state anhedonia. b) State anhedonia partially mediates the associations between PRS for anhedonia and total grey/white matter. **p < .01. ***p < .001. PRS, polygenic risk score.


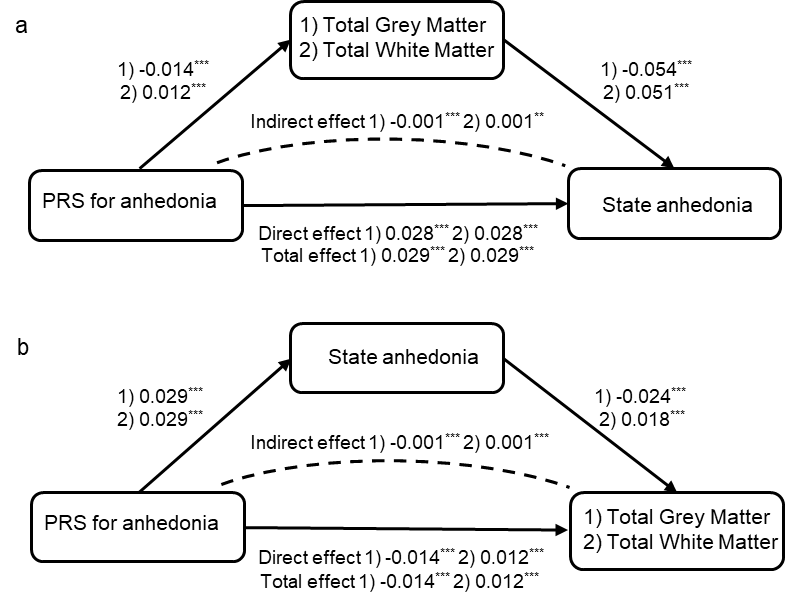


# Supplementary tables

**Table S1. Participant exclusion criteria according to self-reported cancer and non-cancer illness (data field 20001 and 20002).**

| Benign neuroma |
| --- |
| Brain abscess/intracranial abscess |
| Brain cancer/primary malignant brain tumour |
| Brain haemorrhage |
| Cerebral aneurysm |
| Cerebral palsy |
| Chronic/degenerative neurological problem |
| dementia/alzheimers/cognitive impairment |
| Encephalitis |
| Epilepsy |
| Fracture skull/head |
| Head injury |
| Ischaemic stroke |
| Meningeal cancer/malignant meningioma |
| Meningioma/benign meningeal tumour |
| Meningitis |
| Motor Neurone Disease |
| Multiple Sclerosis |
| Nervous system infection |
| Neurological injury/trauma |
| Other demyelinating disease (not Multiple Sclerosis) |
| Other neurological problem |
| Parkinson’s Disease |
| Spina Bifida |
| Stroke |
| Subarachnoid haemorrhage |
| Subdural haemorrhage/haematoma |
| Transient ischaemic attack |

**Table S2. The associations between anhedonia, PRS-anhedonia, brain volumes, cortical thickness and white matter integrity.** The models of anhedonia were conducted with age, age^2^, sex, total brain volume and head position coordinates set as covariates. The models of PRS-anhedonia also included genotype array and the first ten genetic principal components as covariates. Hemisphere was also set as a covariate when appropriate.

| **Outcome** | **State anhedonia** | | | | | |  | **Polygenic risk for anhedonia** | | | | | |
| --- | --- | --- | --- | --- | --- | --- | --- | --- | --- | --- | --- | --- | --- |
|  | **N** | **β** | **SE** | **Z** | **p** | **p_corrected_** |  | **N** | **β** | **SE** | **Z** | **p** | **p_corrected_** |
| Total grey matter volume | 19 564 | -0.025 | 0.005 | -5.380 | <0.001 | <0.001 |  | 16 626 | -0.014 | 0.003 | -5.520 | <0.001 | <0.001 |
| Total white matter volume | 19 553 | 0.017 | 0.004 | 3.940 | <0.001 | <0.001 |  | 16 613 | 0.012 | 0.002 | 5.320 | <0.001 | <0.001 |
| Thalamus | 19 504 | -0.04 | 0.009 | -4.640 | <0.001 | <0.001 |  | 16 574 | <0.001 | 0.005 | 0.080 | 0.933 | 0.968 |
| Caudate | 19 469 | -0.002 | 0.011 | -0.180 | 0.861 | 0.888 |  | 16 545 | -0.012 | 0.006 | -1.930 | 0.054 | 0.157 |
| Putamen | 19 481 | -0.015 | 0.01 | -1.530 | 0.126 | 0.200 |  | 16 555 | -0.004 | 0.006 | -0.690 | 0.492 | 0.694 |
| Pallidum | 19 349 | -0.016 | 0.011 | -1.390 | 0.163 | 0.209 |  | 16 441 | -0.004 | 0.006 | -0.720 | 0.470 | 0.694 |
| Hippocampus | 19 375 | -0.016 | 0.011 | -1.500 | 0.135 | 0.200 |  | 16 464 | -0.006 | 0.006 | -0.920 | 0.358 | 0.551 |
| Amygdala | 19 490 | -0.019 | 0.011 | -1.740 | 0.081 | 0.149 |  | 16 564 | -0.002 | 0.006 | -0.320 | 0.749 | 0.856 |
| Accumbens | 19 497 | -0.051 | 0.011 | -4.640 | <0.001 | <0.001 |  | 16 568 | -0.015 | 0.006 | -2.410 | 0.016 | 0.091 |
| **Cortical thickness** |  |  |  |  |  |  |  |  |  |  |  |  |  |
| Caudal anterior cingulate | 19 164 | -0.022 | 0.011 | -1.930 | 0.053 | 0.128 |  | 16 287 | <0.001 | 0.006 | -0.070 | 0.944 | 0.968 |
| Caudal middle frontal | 19 183 | -0.018 | 0.013 | -1.410 | 0.158 | 0.209 |  | 16 294 | -0.008 | 0.007 | -1.200 | 0.231 | 0.437 |
| Cuneus | 19 274 | 0.018 | 0.013 | 1.390 | 0.166 | 0.209 |  | 16 376 | 0.005 | 0.007 | 0.650 | 0.513 | 0.694 |
| Entorhinal | 19 112 | -0.02 | 0.012 | -1.680 | 0.093 | 0.162 |  | 16 229 | -0.009 | 0.007 | -1.380 | 0.169 | 0.356 |
| Fusiform | 19 225 | -0.019 | 0.012 | -1.530 | 0.125 | 0.200 |  | 16 333 | -0.014 | 0.007 | -2.080 | 0.037 | 0.123 |
| Inferior parietal | 19 135 | -0.015 | 0.013 | -1.140 | 0.252 | 0.305 |  | 16 250 | -0.001 | 0.007 | -0.100 | 0.919 | 0.968 |
| Inferior temporal | 19 222 | -0.027 | 0.013 | -2.180 | 0.030 | 0.095 |  | 16 331 | -0.008 | 0.007 | -1.150 | 0.251 | 0.437 |
| Isthmus cingulate | 19 245 | -0.023 | 0.012 | -1.880 | 0.060 | 0.128 |  | 16 348 | -0.013 | 0.007 | -1.920 | 0.055 | 0.157 |
| Lateral occipital | 19 258 | 0.024 | 0.013 | 1.900 | 0.058 | 0.128 |  | 16 363 | 0.003 | 0.007 | 0.460 | 0.648 | 0.785 |
| Lateral orbitofrontal | 19 258 | -0.018 | 0.013 | -1.450 | 0.147 | 0.209 |  | 16 358 | -0.013 | 0.007 | -1.880 | 0.060 | 0.160 |
| Lingual | 19 275 | 0.022 | 0.013 | 1.740 | 0.082 | 0.149 |  | 16 374 | -0.008 | 0.007 | -1.150 | 0.251 | 0.437 |
| Medial orbitofrontal | 19 251 | -0.023 | 0.012 | -1.870 | 0.061 | 0.128 |  | 16 356 | -0.017 | 0.007 | -2.480 | 0.013 | 0.087 |
| Middle temporal | 19 272 | -0.026 | 0.012 | -2.100 | 0.036 | 0.101 |  | 16 369 | -0.012 | 0.007 | -1.720 | 0.085 | 0.213 |
| Parahippocampal | 19 265 | -0.027 | 0.012 | -2.150 | 0.031 | 0.095 |  | 16 369 | -0.032 | 0.007 | -4.630 | <0.001 | <0.001 |
| Paracentral | 19 243 | -0.04 | 0.013 | -3.140 | 0.002 | 0.010 |  | 16 348 | -0.016 | 0.007 | -2.320 | 0.021 | 0.093 |
| Pars opercularis | 19 160 | -0.04 | 0.012 | -3.180 | 0.001 | 0.007 |  | 16 273 | -0.008 | 0.007 | -1.230 | 0.218 | 0.436 |
| Pars orbitalis | 19 210 | -0.018 | 0.012 | -1.500 | 0.135 | 0.200 |  | 16 320 | -0.014 | 0.007 | -2.150 | 0.032 | 0.116 |
| Pars triangularis | 19 128 | -0.017 | 0.013 | -1.380 | 0.167 | 0.209 |  | 16 242 | -0.007 | 0.007 | -1.030 | 0.304 | 0.507 |
| Pericalcarine | 19 279 | 0.029 | 0.013 | 2.290 | 0.022 | 0.080 |  | 16 379 | <0.001 | 0.007 | 0.040 | 0.969 | 0.969 |
| Postcentral | 19 252 | -0.002 | 0.013 | -0.170 | 0.866 | 0.888 |  | 16 354 | -0.004 | 0.007 | -0.620 | 0.538 | 0.694 |
| Posterior cingulate | 19 266 | -0.012 | 0.012 | -0.980 | 0.327 | 0.385 |  | 16 369 | -0.01 | 0.007 | -1.470 | 0.141 | 0.313 |
| Precentral | 19 185 | -0.033 | 0.013 | -2.570 | 0.010 | 0.044 |  | 16 294 | -0.016 | 0.007 | -2.240 | 0.025 | 0.100 |
| Precuneus | 19 219 | -0.011 | 0.013 | -0.870 | 0.383 | 0.426 |  | 16 323 | 0.002 | 0.007 | 0.320 | 0.747 | 0.856 |
| Rostral anterior cingulate | 19 232 | -0.042 | 0.012 | -3.650 | <0.001 | <0.001 |  | 16 338 | -0.006 | 0.006 | -0.940 | 0.348 | 0.551 |
| Rostral middle frontal | 19 095 | -0.01 | 0.013 | -0.770 | 0.440 | 0.476 |  | 16 221 | -0.004 | 0.007 | -0.520 | 0.602 | 0.753 |
| Superior frontal | 19 183 | -0.026 | 0.013 | -2.070 | 0.038 | 0.101 |  | 16 294 | -0.012 | 0.007 | -1.680 | 0.094 | 0.221 |
| Superior parietal | 19 184 | 0.012 | 0.013 | 0.920 | 0.357 | 0.408 |  | 16 294 | 0.005 | 0.007 | 0.630 | 0.531 | 0.694 |
| Superior temporal | 19 248 | -0.03 | 0.012 | -2.430 | 0.015 | 0.060 |  | 16 350 | -0.024 | 0.007 | -3.470 | 0.001 | 0.008 |
| Supramarginal | 19 157 | -0.023 | 0.013 | -1.820 | 0.069 | 0.138 |  | 16 272 | -0.001 | 0.007 | -0.110 | 0.915 | 0.968 |
| Transverse temporal | 19 298 | 0.001 | 0.013 | 0.040 | 0.968 | 0.968 |  | 16 393 | -0.016 | 0.007 | -2.320 | 0.021 | 0.093 |
| Insula | 19 250 | -0.038 | 0.013 | -3.050 | 0.002 | 0.010 |  | 16 353 | -0.03 | 0.007 | -4.340 | <0.001 | <0.001 |
| **Fractional anisotropy** |  |  |  |  |  |  |  |  |  |  |  |  |  |
| Forceps major | 17 602 | -0.043 | 0.015 | -2.890 | 0.004 | 0.010 |  | 14 945 | -0.016 | 0.008 | -1.910 | 0.057 | 0.107 |
| Forceps minor | 17 680 | -0.027 | 0.014 | -1.920 | 0.055 | 0.094 |  | 15 008 | -0.022 | 0.008 | -2.780 | 0.005 | 0.021 |
| Middle cerebellar peduncle | 17 556 | 0.012 | 0.015 | 0.780 | 0.434 | 0.476 |  | 14 911 | 0.005 | 0.008 | 0.680 | 0.498 | 0.623 |
| Acoustic radiation | 17 678 | -0.015 | 0.013 | -1.150 | 0.251 | 0.341 |  | 15 012 | 0.014 | 0.007 | 1.970 | 0.049 | 0.098 |
| Anterior thalamic radiation | 17 652 | -0.039 | 0.014 | -2.820 | 0.005 | 0.012 |  | 14 986 | -0.014 | 0.008 | -1.780 | 0.076 | 0.127 |
| Cingulate gyrus part of cingulum | 17 716 | 0.01 | 0.013 | 0.820 | 0.413 | 0.476 |  | 15 041 | 0.002 | 0.007 | 0.270 | 0.784 | 0.784 |
| Parahippocampal part of cingulum | 17 307 | 0.018 | 0.013 | 1.400 | 0.162 | 0.239 |  | 14 701 | 0.009 | 0.007 | 1.290 | 0.196 | 0.267 |
| Corticospinal tract | 17 667 | -0.004 | 0.014 | -0.320 | 0.748 | 0.771 |  | 15 005 | -0.003 | 0.007 | -0.470 | 0.641 | 0.712 |
| Inferior fronto-occipital fasciculus | 17 678 | -0.025 | 0.014 | -1.780 | 0.074 | 0.120 |  | 15 010 | -0.014 | 0.008 | -1.800 | 0.072 | 0.127 |
| Inferior longitudinal fasciculus | 17 656 | -0.038 | 0.014 | -2.740 | 0.006 | 0.014 |  | 14 990 | -0.004 | 0.008 | -0.510 | 0.611 | 0.705 |
| Medial lemniscus | 17 647 | -0.013 | 0.012 | -1.060 | 0.290 | 0.379 |  | 14 992 | -0.011 | 0.007 | -1.660 | 0.096 | 0.152 |
| Posterior thalamic radiation | 17 628 | -0.055 | 0.014 | -4.020 | <0.001 | <0.001 |  | 14 968 | -0.02 | 0.008 | -2.730 | 0.006 | 0.023 |
| Superior longitudinal fasciculus | 17 664 | -0.036 | 0.014 | -2.540 | 0.011 | 0.022 |  | 14 998 | -0.011 | 0.008 | -1.480 | 0.139 | 0.209 |
| Superior thalamic radiation | 17 645 | -0.009 | 0.014 | -0.610 | 0.542 | 0.576 |  | 14 987 | -0.011 | 0.008 | -1.380 | 0.167 | 0.239 |
| Uncinate fasciculus | 17 694 | -0.012 | 0.013 | -0.970 | 0.330 | 0.401 |  | 15 021 | -0.004 | 0.007 | -0.590 | 0.557 | 0.668 |
| **Mean diffusivity** |  |  |  |  |  |  |  |  |  |  |  |  |  |
| Forceps major | 17 567 | 0.045 | 0.014 | 3.150 | 0.002 | 0.006 |  | 14 916 | 0.009 | 0.008 | 1.150 | 0.249 | 0.325 |
| Forceps minor | 17 623 | 0.037 | 0.014 | 2.660 | 0.008 | 0.017 |  | 14 965 | 0.023 | 0.008 | 3.000 | 0.003 | 0.021 |
| Middle cerebellar peduncle | 17 585 | 0.025 | 0.014 | 1.740 | 0.082 | 0.127 |  | 14 935 | 0.025 | 0.008 | 3.110 | 0.002 | 0.021 |
| Acoustic radiation | 17 590 | 0.013 | 0.013 | 1.020 | 0.308 | 0.388 |  | 14 939 | 0.014 | 0.007 | 1.980 | 0.048 | 0.098 |
| Acoustic radiation (left) | 17 603 | 0.035 | 0.015 | 2.400 | 0.016 | 0.030 |  | — | — | — | — | — | — |
| Acoustic radiation (right) | 17 577 | -0.012 | 0.015 | -0.800 | 0.421 | 0.476 |  | — | — | — | — | — | — |
| Anterior thalamic radiation | 17 437 | 0.064 | 0.013 | 5.040 | <0.001 | <0.001 |  | 14 937 | 0.019 | 0.007 | 2.700 | 0.007 | 0.023 |
| Cingulate gyrus part of cingulum | 17 596 | 0.052 | 0.013 | 3.910 | <0.001 | <0.001 |  | 14 939 | 0.022 | 0.007 | 2.990 | 0.003 | 0.021 |
| Parahippocampal part of cingulum | 17 138 | 0.015 | 0.013 | 1.210 | 0.224 | 0.317 |  | 14 556 | -0.002 | 0.007 | -0.330 | 0.745 | 0.784 |
| Corticospinal tract | 17 621 | 0.051 | 0.014 | 3.720 | <0.001 | <0.001 |  | 14 966 | 0.017 | 0.008 | 2.210 | 0.027 | 0.062 |
| Inferior fronto-occipital fasciculus | 17 598 | 0.049 | 0.013 | 3.680 | <0.001 | <0.001 |  | 14 938 | 0.021 | 0.007 | 2.840 | 0.005 | 0.021 |
| Inferior longitudinal fasciculus | 17 577 | 0.045 | 0.013 | 3.390 | 0.001 | 0.003 |  | 14 917 | 0.018 | 0.007 | 2.430 | 0.015 | 0.038 |
| Medial lemniscus | 17 636 | -0.002 | 0.013 | -0.180 | 0.857 | 0.857 |  | 14 977 | -0.002 | 0.007 | -0.290 | 0.775 | 0.784 |
| Posterior thalamic radiation | 17 376 | 0.046 | 0.013 | 3.550 | <0.001 | <0.001 |  | 14 746 | 0.027 | 0.007 | 3.730 | <0.001 | <0.001 |
| Superior longitudinal fasciculus | 17 505 | 0.064 | 0.014 | 4.550 | <0.001 | <0.001 |  | 14 855 | 0.022 | 0.008 | 2.910 | 0.004 | 0.021 |
| Superior thalamic radiation | 17 440 | 0.066 | 0.013 | 5.270 | <0.001 | <0.001 |  | 14 804 | 0.018 | 0.007 | 2.620 | 0.009 | 0.027 |
| Uncinate fasciculus | 17 626 | 0.042 | 0.012 | 3.380 | 0.001 | 0.003 |  | 14 966 | 0.017 | 0.007 | 2.490 | 0.013 | 0.035 |
| Uncinate fasciculus (left) | 17 632 | 0.029 | 0.013 | 2.240 | 0.025 | 0.045 |  | — | — | — | — | — | — |
| Uncinate fasciculus (right) | 17 619 | 0.056 | 0.014 | 4.080 | <0.001 | <0.001 |  | — | — | — | — | — | — |

**Table S3. The interaction between anhedonia, PRS-anhedonia and hemisphere on the subcortical volumes, whole-surface cortical thickness and white matter integrity.** In all models, age, age^2^, sex, total ICV, and lateral, transverse and longitudinal scanner position coordinates were set as covariates. For PRS-anhedonia, genotype array and the first ten genetic principal components were set as covariates additionally. Anhedonia/PRS-anhedonia, hemisphere and the interaction between anhedonia/PRS-anhedonia and hemisphere were also included in the model. As total grey/white matter volume were whole-brain measures, and forceps major, forceps minor and middle cerebellar peduncle were single tracts, these measures were not tested in this model. Significant interaction with anhedonia were found in FA of uncinate fasciculus, MD of acoustic radiation and uncinate fasciculus, therefore individual tests on the value of each hemisphere were conducted for anhedonia and the results were added in Table S3.

| **Outcome** | **State anhedonia** | | | | |  | **Polygenic risk for anhedonia** | | | | |
| --- | --- | --- | --- | --- | --- | --- | --- | --- | --- | --- | --- |
|  | **β** | **SE** | **Z** | **p** | **p_corrected_** |  | **β** | **SE** | **Z** | **p** | **p_corrected_** |
| Thalamus | -0.002 | 0.005 | -0.310 | 0.755 | 0.956 |  | <0.001 | 0.003 | 0.050 | 0.963 | 0.963 |
| Caudate | -0.007 | 0.006 | -1.100 | 0.271 | 0.736 |  | -0.003 | 0.004 | -0.790 | 0.431 | 0.953 |
| Putamen | -0.007 | 0.008 | -0.920 | 0.359 | 0.778 |  | 0.008 | 0.004 | 2.030 | 0.042 | 0.504 |
| Pallidum | 0.013 | 0.010 | 1.260 | 0.208 | 0.736 |  | -0.005 | 0.006 | -0.860 | 0.392 | 0.931 |
| Hippocampus | 0.001 | 0.012 | 0.090 | 0.927 | 0.980 |  | 0.015 | 0.007 | 2.140 | 0.032 | 0.504 |
| Amygdala | 0.012 | 0.016 | 0.790 | 0.430 | 0.778 |  | 0.017 | 0.009 | 1.940 | 0.053 | 0.504 |
| Accumbens | 0.006 | 0.013 | 0.440 | 0.660 | 0.956 |  | -0.003 | 0.007 | -0.440 | 0.66 | 0.953 |
| **Cortical thickness** |  |  |  |  |  |  |  |  |  |  |  |
| Caudal anterior cingulate | 0.006 | 0.017 | 0.320 | 0.746 | 0.956 |  | 0.005 | 0.009 | 0.500 | 0.619 | 0.953 |
| Caudal middle frontal | -0.013 | 0.011 | -1.140 | 0.254 | 0.736 |  | -0.003 | 0.006 | -0.490 | 0.625 | 0.953 |
| Cuneus | -0.004 | 0.013 | -0.340 | 0.735 | 0.956 |  | -0.007 | 0.007 | -1.030 | 0.304 | 0.825 |
| Entorhinal | 0.004 | 0.016 | 0.260 | 0.798 | 0.971 |  | -0.001 | 0.009 | -0.100 | 0.922 | 0.963 |
| Fusiform | 0.029 | 0.013 | 2.190 | 0.028 | 0.736 |  | 0.01 | 0.007 | 1.320 | 0.187 | 0.825 |
| Inferior parietal | 0.006 | 0.011 | 0.520 | 0.601 | 0.956 |  | -0.007 | 0.006 | -1.180 | 0.237 | 0.825 |
| Inferior temporal | 0.018 | 0.014 | 1.300 | 0.192 | 0.736 |  | -0.002 | 0.008 | -0.270 | 0.787 | 0.953 |
| Isthmus cingulate | 0.013 | 0.014 | 0.880 | 0.378 | 0.778 |  | 0.002 | 0.008 | 0.220 | 0.825 | 0.953 |
| Lateral occipital | -0.002 | 0.012 | -0.170 | 0.865 | 0.980 |  | -0.001 | 0.007 | -0.210 | 0.832 | 0.953 |
| Lateral orbitofrontal | 0.018 | 0.014 | 1.340 | 0.179 | 0.736 |  | -0.008 | 0.007 | -1.030 | 0.303 | 0.825 |
| Lingual | -0.001 | 0.013 | -0.090 | 0.928 | 0.980 |  | 0.003 | 0.007 | 0.450 | 0.651 | 0.953 |
| Medial orbitofrontal | 0.013 | 0.016 | 0.810 | 0.419 | 0.778 |  | -0.003 | 0.009 | -0.340 | 0.732 | 0.953 |
| Middle temporal | 0.017 | 0.014 | 1.240 | 0.214 | 0.736 |  | -0.003 | 0.008 | -0.370 | 0.712 | 0.953 |
| Parahippocampal | -0.015 | 0.014 | -1.120 | 0.264 | 0.736 |  | 0.01 | 0.008 | 1.250 | 0.212 | 0.825 |
| Paracentral | -0.003 | 0.011 | -0.310 | 0.753 | 0.956 |  | -0.007 | 0.006 | -1.160 | 0.248 | 0.825 |
| Pars opercularis | 0.003 | 0.013 | 0.230 | 0.818 | 0.971 |  | -0.002 | 0.007 | -0.290 | 0.775 | 0.953 |
| Pars orbitalis | 0.021 | 0.015 | 1.430 | 0.154 | 0.736 |  | <0.001 | 0.008 | 0.050 | 0.960 | 0.963 |
| Pars triangularis | 0.002 | 0.013 | 0.130 | 0.893 | 0.980 |  | -0.002 | 0.007 | -0.330 | 0.744 | 0.953 |
| Pericalcarine | -0.004 | 0.014 | -0.320 | 0.746 | 0.956 |  | 0.001 | 0.008 | 0.110 | 0.911 | 0.963 |
| Postcentral | <0.001 | 0.012 | 0.000 | 0.997 | 0.997 |  | -0.006 | 0.007 | -0.920 | 0.358 | 0.907 |
| Posterior cingulate | 0.012 | 0.015 | 0.790 | 0.429 | 0.778 |  | -0.017 | 0.008 | -2.010 | 0.045 | 0.504 |
| Precentral | 0.016 | 0.011 | 1.490 | 0.136 | 0.736 |  | -0.008 | 0.006 | -1.340 | 0.180 | 0.825 |
| Precuneus | 0.009 | 0.010 | 0.860 | 0.389 | 0.778 |  | -0.006 | 0.005 | -1.090 | 0.275 | 0.825 |
| Rostral anterior cingulate | -0.008 | 0.016 | -0.520 | 0.605 | 0.956 |  | 0.004 | 0.009 | 0.500 | 0.620 | 0.953 |
| Rostral middle frontal | -0.001 | 0.011 | -0.050 | 0.957 | 0.983 |  | -0.002 | 0.006 | -0.390 | 0.698 | 0.953 |
| Superior frontal | 0.011 | 0.010 | 1.100 | 0.270 | 0.736 |  | -0.006 | 0.005 | -1.130 | 0.26 | 0.825 |
| Superior parietal | 0.020 | 0.010 | 1.950 | 0.051 | 0.736 |  | 0.004 | 0.006 | 0.630 | 0.529 | 0.953 |
| Superior temporal | 0.023 | 0.012 | 1.890 | 0.059 | 0.736 |  | -0.004 | 0.007 | -0.640 | 0.522 | 0.953 |
| Supramarginal | 0.006 | 0.012 | 0.520 | 0.602 | 0.956 |  | -0.007 | 0.006 | -1.150 | 0.249 | 0.825 |
| Transverse temporal | 0.012 | 0.014 | 0.830 | 0.406 | 0.778 |  | -0.001 | 0.008 | -0.190 | 0.853 | 0.953 |
| Insula | 0.019 | 0.013 | 1.420 | 0.155 | 0.736 |  | 0.002 | 0.007 | 0.290 | 0.769 | 0.953 |
| **Fractional anisotropy** |  |  |  |  |  |  |  |  |  |  |  |
| Acoustic radiation | -0.021 | 0.014 | -1.460 | 0.143 | 0.312 |  | 0.002 | 0.008 | 0.250 | 0.801 | 0.987 |
| Anterior thalamic radiation | -0.004 | 0.009 | -0.410 | 0.681 | 0.711 |  | -0.002 | 0.005 | -0.450 | 0.652 | 0.987 |
| Cingulate gyrus part of cingulum | -0.027 | 0.014 | -1.920 | 0.055 | 0.183 |  | <0.001 | 0.008 | 0.020 | 0.985 | 0.987 |
| Parahippocampal part of cingulum | -0.027 | 0.015 | -1.880 | 0.061 | 0.183 |  | 0.003 | 0.008 | 0.420 | 0.671 | 0.987 |
| Corticospinal tract | -0.018 | 0.010 | -1.760 | 0.078 | 0.208 |  | 0.002 | 0.006 | 0.380 | 0.706 | 0.987 |
| Inferior fronto-occipital fasciculus | -0.021 | 0.009 | -2.410 | 0.016 | 0.077 |  | 0.002 | 0.005 | 0.390 | 0.698 | 0.987 |
| Inferior longitudinal fasciculus | -0.008 | 0.008 | -1.000 | 0.320 | 0.515 |  | <0.001 | 0.005 | 0.020 | 0.986 | 0.987 |
| Medial lemniscus | -0.038 | 0.015 | -2.440 | 0.015 | 0.077 |  | -0.018 | 0.008 | -2.110 | 0.035 | 0.84 |
| Posterior thalamic radiation | -0.013 | 0.010 | -1.300 | 0.193 | 0.356 |  | -0.003 | 0.006 | -0.540 | 0.588 | 0.987 |
| Superior longitudinal fasciculus | 0.004 | 0.008 | 0.540 | 0.588 | 0.685 |  | -0.003 | 0.004 | -0.660 | 0.512 | 0.987 |
| Superior thalamic radiation | -0.011 | 0.008 | -1.390 | 0.166 | 0.332 |  | -0.002 | 0.004 | -0.470 | 0.639 | 0.987 |
| Uncinate fasciculus | -0.034 | 0.013 | -2.650 | 0.008 | 0.064 |  | -0.005 | 0.007 | -0.770 | 0.44 | 0.987 |
| **Mean diffusivity** |  |  |  |  |  |  |  |  |  |  |  |
| Acoustic radiation | -0.050 | 0.015 | -3.240 | 0.001 | 0.012 |  | 0.007 | 0.009 | 0.820 | 0.413 | 0.987 |
| Anterior thalamic radiation | 0.014 | 0.007 | 1.970 | 0.049 | 0.183 |  | <0.001 | 0.004 | 0.100 | 0.919 | 0.987 |
| Cingulate gyrus part of cingulum | 0.005 | 0.009 | 0.510 | 0.610 | 0.685 |  | -0.003 | 0.005 | -0.590 | 0.558 | 0.987 |
| Parahippocampal part of cingulum | 0.011 | 0.015 | 0.750 | 0.453 | 0.604 |  | -0.006 | 0.008 | -0.690 | 0.49 | 0.987 |
| Corticospinal tract | 0.010 | 0.012 | 0.900 | 0.367 | 0.544 |  | -0.001 | 0.006 | -0.150 | 0.881 | 0.987 |
| Inferior fronto-occipital fasciculus | 0.013 | 0.008 | 1.680 | 0.093 | 0.223 |  | 0.003 | 0.004 | 0.620 | 0.537 | 0.987 |
| Inferior longitudinal fasciculus | 0.003 | 0.007 | 0.480 | 0.628 | 0.685 |  | <0.001 | 0.004 | 0.090 | 0.927 | 0.987 |
| Medial lemniscus | 0.014 | 0.015 | 0.990 | 0.322 | 0.515 |  | <0.001 | 0.008 | 0.050 | 0.958 | 0.987 |
| Posterior thalamic radiation | 0.007 | 0.009 | 0.690 | 0.489 | 0.618 |  | <0.001 | 0.005 | -0.020 | 0.987 | 0.987 |
| Superior longitudinal fasciculus | -0.001 | 0.006 | -0.220 | 0.827 | 0.827 |  | 0.003 | 0.003 | 0.900 | 0.369 | 0.987 |
| Superior thalamic radiation | 0.006 | 0.007 | 0.870 | 0.385 | 0.544 |  | 0.001 | 0.004 | 0.130 | 0.894 | 0.987 |
| Uncinate fasciculus | 0.032 | 0.010 | 3.320 | 0.001 | 0.012 |  | 0.007 | 0.005 | 1.290 | 0.197 | 0.987 |

**Table S4. The associations between anhedonia, PRS-anhedonia, brain volumes, cortical thickness and white matter integrity.** The models of anhedonia were conducted with age, age^2^, sex, total brain volume, head position coordinates and assessment center set as covariates. The models of PRS-anhedonia also included genotype array and the first ten genetic principal components as covariates. Hemisphere was also set as a covariate when appropriate.

| **Outcome** | **State anhedonia** | | | | |  | **Polygenic risk for anhedonia** | | | | |
| --- | --- | --- | --- | --- | --- | --- | --- | --- | --- | --- | --- |
|  | **β** | **SE** | **Z** | **p** | **p_corrected_** |  | **β** | **SE** | **Z** | **p** | **p_corrected_** |
| Total grey matter volume | -0.025 | 0.005 | -5.370 | <0.001 | <0.001 |  | -0.014 | 0.003 | -5.490 | <0.001 | <0.001 |
| Total white matter volume | 0.017 | 0.004 | 3.940 | <0.001 | <0.001 |  | 0.012 | 0.002 | 5.330 | <0.001 | <0.001 |
| Thalamus | -0.040 | 0.009 | -4.630 | <0.001 | <0.001 |  | 0.001 | 0.005 | 0.140 | 0.885 | 0.931 |
| Caudate | -0.002 | 0.011 | -0.190 | 0.846 | 0.891 |  | -0.013 | 0.006 | -2.010 | 0.045 | 0.153 |
| Putamen | -0.015 | 0.010 | -1.550 | 0.122 | 0.203 |  | -0.004 | 0.006 | -0.740 | 0.459 | 0.644 |
| Pallidum | -0.016 | 0.011 | -1.400 | 0.162 | 0.214 |  | -0.004 | 0.006 | -0.730 | 0.467 | 0.644 |
| Hippocampus | -0.017 | 0.011 | -1.510 | 0.130 | 0.208 |  | -0.006 | 0.006 | -0.970 | 0.332 | 0.553 |
| Amygdala | -0.019 | 0.011 | -1.780 | 0.075 | 0.143 |  | -0.003 | 0.006 | -0.460 | 0.647 | 0.784 |
| Accumbens | -0.051 | 0.011 | -4.640 | <0.001 | <0.001 |  | -0.015 | 0.006 | -2.420 | 0.015 | 0.100 |
| **Cortical thickness** |  |  |  |  |  |  |  |  |  |  |  |
| Caudal anterior cingulate | -0.022 | 0.011 | -1.900 | 0.058 | 0.136 |  | <0.001 | 0.006 | 0.070 | 0.944 | 0.944 |
| Caudal middle frontal | -0.017 | 0.013 | -1.390 | 0.166 | 0.214 |  | -0.007 | 0.007 | -1.030 | 0.303 | 0.537 |
| Cuneus | 0.018 | 0.013 | 1.390 | 0.165 | 0.214 |  | 0.005 | 0.007 | 0.690 | 0.491 | 0.655 |
| Entorhinal | -0.020 | 0.012 | -1.690 | 0.091 | 0.158 |  | -0.010 | 0.007 | -1.440 | 0.151 | 0.336 |
| Fusiform | -0.019 | 0.012 | -1.500 | 0.135 | 0.208 |  | -0.013 | 0.007 | -1.920 | 0.055 | 0.169 |
| Inferior parietal | -0.014 | 0.013 | -1.110 | 0.267 | 0.324 |  | 0.001 | 0.007 | 0.120 | 0.908 | 0.931 |
| Inferior temporal | -0.027 | 0.012 | -2.140 | 0.032 | 0.098 |  | -0.007 | 0.007 | -1.020 | 0.309 | 0.537 |
| Isthmus cingulate | -0.023 | 0.012 | -1.870 | 0.061 | 0.136 |  | -0.012 | 0.007 | -1.860 | 0.063 | 0.180 |
| Lateral occipital | 0.024 | 0.013 | 1.890 | 0.059 | 0.136 |  | 0.003 | 0.007 | 0.420 | 0.677 | 0.796 |
| Lateral orbitofrontal | -0.018 | 0.013 | -1.420 | 0.156 | 0.214 |  | -0.012 | 0.007 | -1.710 | 0.087 | 0.232 |
| Lingual | 0.022 | 0.013 | 1.740 | 0.081 | 0.147 |  | -0.008 | 0.007 | -1.140 | 0.256 | 0.512 |
| Medial orbitofrontal | -0.022 | 0.012 | -1.840 | 0.066 | 0.139 |  | -0.015 | 0.007 | -2.310 | 0.021 | 0.116 |
| Middle temporal | -0.025 | 0.012 | -2.050 | 0.040 | 0.107 |  | -0.01 | 0.007 | -1.520 | 0.128 | 0.306 |
| Parahippocampal | -0.026 | 0.012 | -2.140 | 0.032 | 0.098 |  | -0.031 | 0.007 | -4.600 | <0.001 | <0.001 |
| Paracentral | -0.040 | 0.013 | -3.130 | 0.002 | 0.011 |  | -0.016 | 0.007 | -2.240 | 0.025 | 0.116 |
| Pars opercularis | -0.039 | 0.012 | -3.150 | 0.002 | 0.011 |  | -0.007 | 0.007 | -1.020 | 0.308 | 0.537 |
| Pars orbitalis | -0.018 | 0.012 | -1.470 | 0.142 | 0.210 |  | -0.013 | 0.007 | -2.000 | 0.046 | 0.153 |
| Pars triangularis | -0.017 | 0.013 | -1.350 | 0.177 | 0.221 |  | -0.006 | 0.007 | -0.850 | 0.394 | 0.623 |
| Pericalcarine | 0.029 | 0.013 | 2.320 | 0.020 | 0.073 |  | 0.001 | 0.007 | 0.150 | 0.881 | 0.931 |
| Postcentral | -0.002 | 0.013 | -0.150 | 0.884 | 0.907 |  | -0.004 | 0.007 | -0.500 | 0.614 | 0.768 |
| Posterior cingulate | -0.011 | 0.012 | -0.950 | 0.341 | 0.390 |  | -0.009 | 0.007 | -1.350 | 0.178 | 0.375 |
| Precentral | -0.033 | 0.013 | -2.560 | 0.010 | 0.044 |  | -0.015 | 0.007 | -2.160 | 0.031 | 0.124 |
| Precuneus | -0.011 | 0.013 | -0.840 | 0.403 | 0.448 |  | 0.004 | 0.007 | 0.520 | 0.605 | 0.768 |
| Rostral anterior cingulate | -0.042 | 0.012 | -3.630 | <0.001 | <0.001 |  | -0.005 | 0.006 | -0.830 | 0.405 | 0.623 |
| Rostral middle frontal | -0.010 | 0.013 | -0.750 | 0.455 | 0.492 |  | -0.003 | 0.007 | -0.370 | 0.714 | 0.816 |
| Superior frontal | -0.026 | 0.013 | -2.050 | 0.040 | 0.107 |  | -0.011 | 0.007 | -1.510 | 0.130 | 0.306 |
| Superior parietal | 0.012 | 0.013 | 0.960 | 0.339 | 0.390 |  | 0.006 | 0.007 | 0.800 | 0.426 | 0.631 |
| Superior temporal | -0.030 | 0.012 | -2.400 | 0.016 | 0.064 |  | -0.023 | 0.007 | -3.300 | 0.001 | 0.008 |
| Supramarginal | -0.023 | 0.013 | -1.780 | 0.075 | 0.143 |  | 0.001 | 0.007 | 0.120 | 0.903 | 0.931 |
| Transverse temporal | 0.001 | 0.013 | 0.060 | 0.951 | 0.951 |  | -0.015 | 0.007 | -2.220 | 0.026 | 0.116 |
| Insula | -0.038 | 0.013 | -3.020 | 0.003 | 0.015 |  | -0.029 | 0.007 | -4.200 | <0.001 | <0.001 |
| **Fractional anisotropy** |  |  |  |  |  |  |  |  |  |  |  |
| Forceps major | -0.044 | 0.015 | -2.970 | 0.003 | 0.008 |  | -0.017 | 0.008 | -2.140 | 0.032 | 0.074 |
| Forceps minor | -0.028 | 0.014 | -1.930 | 0.054 | 0.092 |  | -0.022 | 0.008 | -2.840 | 0.005 | 0.021 |
| Middle cerebellar peduncle | 0.011 | 0.015 | 0.730 | 0.464 | 0.509 |  | 0.004 | 0.008 | 0.530 | 0.594 | 0.636 |
| Acoustic radiation | -0.016 | 0.013 | -1.260 | 0.209 | 0.296 |  | 0.012 | 0.007 | 1.650 | 0.098 | 0.163 |
| Anterior thalamic radiation | -0.039 | 0.014 | -2.850 | 0.004 | 0.010 |  | -0.015 | 0.008 | -1.910 | 0.056 | 0.105 |
| Cingulate gyrus part of cingulum | 0.010 | 0.013 | 0.790 | 0.427 | 0.496 |  | 0.001 | 0.007 | 0.190 | 0.846 | 0.846 |
| Parahippocampal part of cingulum | 0.018 | 0.013 | 1.390 | 0.164 | 0.242 |  | 0.009 | 0.007 | 1.230 | 0.220 | 0.287 |
| Corticospinal tract | -0.006 | 0.013 | -0.440 | 0.656 | 0.656 |  | -0.006 | 0.007 | -0.850 | 0.394 | 0.455 |
| Inferior fronto-occipital fasciculus | -0.025 | 0.014 | -1.800 | 0.072 | 0.117 |  | -0.014 | 0.008 | -1.840 | 0.065 | 0.115 |
| Inferior longitudinal fasciculus | -0.039 | 0.014 | -2.760 | 0.006 | 0.014 |  | -0.005 | 0.008 | -0.600 | 0.551 | 0.612 |
| Medial lemniscus | -0.011 | 0.012 | -0.920 | 0.358 | 0.435 |  | -0.009 | 0.007 | -1.260 | 0.206 | 0.281 |
| Posterior thalamic radiation | -0.054 | 0.014 | -3.970 | <0.001 | <0.001 |  | -0.019 | 0.008 | -2.510 | 0.012 | 0.036 |
| Superior longitudinal fasciculus | -0.036 | 0.014 | -2.550 | 0.011 | 0.022 |  | -0.012 | 0.008 | -1.520 | 0.129 | 0.204 |
| Superior thalamic radiation | -0.009 | 0.014 | -0.630 | 0.530 | 0.563 |  | -0.012 | 0.008 | -1.470 | 0.142 | 0.213 |
| Uncinate fasciculus | -0.014 | 0.013 | -1.080 | 0.281 | 0.367 |  | -0.006 | 0.007 | -0.910 | 0.363 | 0.436 |
| **Mean diffusivity** |  |  |  |  |  |  |  |  |  |  |  |
| Forceps major | 0.046 | 0.014 | 3.230 | 0.001 | 0.003 |  | 0.011 | 0.008 | 1.350 | 0.176 | 0.251 |
| Forceps minor | 0.036 | 0.014 | 2.610 | 0.009 | 0.019 |  | 0.022 | 0.008 | 2.880 | 0.004 | 0.021 |
| Middle cerebellar peduncle | 0.024 | 0.014 | 1.670 | 0.095 | 0.147 |  | 0.023 | 0.008 | 2.860 | 0.004 | 0.021 |
| Acoustic radiation | 0.013 | 0.013 | 1.040 | 0.298 | 0.375 |  | 0.014 | 0.007 | 2.040 | 0.042 | 0.084 |
| Acoustic radiation (left) | 0.036 | 0.015 | 2.410 | 0.016 | 0.030 |  | — | — | — | — | — |
| Acoustic radiation (right) | -0.011 | 0.015 | -0.780 | 0.438 | 0.496 |  | — | — | — | — | — |
| Anterior thalamic radiation | 0.064 | 0.013 | 5.070 | <0.001 | <0.001 |  | 0.020 | 0.007 | 2.800 | 0.005 | 0.021 |
| Cingulate gyrus part of cingulum | 0.052 | 0.013 | 3.880 | <0.001 | <0.001 |  | 0.022 | 0.007 | 2.940 | 0.003 | 0.021 |
| Parahippocampal part of cingulum | 0.015 | 0.013 | 1.220 | 0.224 | 0.305 |  | -0.002 | 0.007 | -0.300 | 0.768 | 0.794 |
| Corticospinal tract | 0.050 | 0.014 | 3.690 | <0.001 | <0.001 |  | 0.016 | 0.008 | 2.100 | 0.036 | 0.077 |
| Inferior fronto-occipital fasciculus | 0.049 | 0.013 | 3.640 | <0.001 | <0.001 |  | 0.020 | 0.007 | 2.720 | 0.007 | 0.026 |
| Inferior longitudinal fasciculus | 0.045 | 0.013 | 3.360 | 0.001 | 0.003 |  | 0.017 | 0.007 | 2.320 | 0.020 | 0.050 |
| Medial lemniscus | -0.006 | 0.012 | -0.480 | 0.629 | 0.648 |  | -0.008 | 0.007 | -1.110 | 0.266 | 0.333 |
| Posterior thalamic radiation | 0.044 | 0.013 | 3.430 | 0.001 | 0.003 |  | 0.023 | 0.007 | 3.230 | 0.001 | 0.021 |
| Superior longitudinal fasciculus | 0.062 | 0.014 | 4.480 | <0.001 | <0.001 |  | 0.020 | 0.008 | 2.650 | 0.008 | 0.027 |
| Superior thalamic radiation | 0.065 | 0.013 | 5.210 | <0.001 | <0.001 |  | 0.016 | 0.007 | 2.370 | 0.018 | 0.049 |
| Uncinate fasciculus | 0.043 | 0.012 | 3.490 | <0.001 | <0.001 |  | 0.019 | 0.007 | 2.820 | 0.005 | 0.021 |
| Uncinate fasciculus (left) | 0.030 | 0.013 | 2.310 | 0.021 | 0.038 |  | — | — | — | — | — |
| Uncinate fasciculus (right) | 0.058 | 0.014 | 4.220 | <0.001 | <0.001 |  | — | — | — | — | — |

**Table S5. The interaction between anhedonia, PRS-anhedonia, and the assessment center on brain volumes, cortical thickness and white matter integrity.** The models of anhedonia were conducted with age, age2, sex, total brain volume and head position coordinates set as covariates. The models of PRS-anhedonia also included genotype array and the first ten genetic principal components as covariates. Hemisphere was also set as a covariate when appropriate.

| **Outcome** | **State anhedonia** | | | | |  | **Polygenic risk for anhedonia** | | | | |
| --- | --- | --- | --- | --- | --- | --- | --- | --- | --- | --- | --- |
|  | **β** | **SE** | **Z** | **p** | **p_corrected_** |  | **β** | **SE** | **Z** | **p** | **p_corrected_** |
| Total grey matter volume | 0.014 | 0.012 | 1.130 | 0.260 | 0.954 |  | -0.003 | 0.007 | -0.370 | 0.710 | 0.978 |
| Total white matter volume | -0.006 | 0.011 | -0.570 | 0.571 | 0.954 |  | 0.003 | 0.006 | 0.460 | 0.645 | 0.978 |
| Thalamus | 0.021 | 0.023 | 0.910 | 0.365 | 0.954 |  | 0.006 | 0.013 | 0.470 | 0.641 | 0.978 |
| Caudate | 0.047 | 0.030 | 1.580 | 0.115 | 0.954 |  | 0.003 | 0.017 | 0.150 | 0.884 | 0.978 |
| Putamen | 0.026 | 0.026 | 0.990 | 0.320 | 0.954 |  | 0.005 | 0.015 | 0.330 | 0.738 | 0.978 |
| Pallidum | 0.018 | 0.029 | 0.630 | 0.530 | 0.954 |  | -0.020 | 0.017 | -1.150 | 0.250 | 0.978 |
| Hippocampus | 0.037 | 0.029 | 1.260 | 0.208 | 0.954 |  | 0.004 | 0.017 | 0.260 | 0.794 | 0.978 |
| Amygdala | -0.025 | 0.029 | -0.850 | 0.394 | 0.954 |  | -0.025 | 0.017 | -1.480 | 0.140 | 0.978 |
| Accumbens | 0.009 | 0.029 | 0.330 | 0.743 | 0.954 |  | -0.011 | 0.017 | -0.660 | 0.512 | 0.978 |
| **Cortical thickness** |  |  |  |  |  |  |  |  |  |  |  |
| Caudal anterior cingulate | -0.005 | 0.030 | -0.170 | 0.864 | 0.974 |  | -0.026 | 0.017 | -1.520 | 0.129 | 0.978 |
| Caudal middle frontal | <0.001 | 0.033 | <0.001 | 0.998 | 0.998 |  | -0.029 | 0.019 | -1.510 | 0.130 | 0.978 |
| Cuneus | -0.007 | 0.034 | -0.210 | 0.836 | 0.974 |  | 0.013 | 0.020 | 0.680 | 0.499 | 0.978 |
| Entorhinal | 0.010 | 0.032 | 0.310 | 0.754 | 0.954 |  | -0.017 | 0.019 | -0.920 | 0.356 | 0.978 |
| Fusiform | -0.018 | 0.033 | -0.560 | 0.575 | 0.954 |  | 0.003 | 0.019 | 0.180 | 0.859 | 0.978 |
| Inferior parietal | 0.002 | 0.033 | 0.050 | 0.960 | 0.985 |  | -0.011 | 0.020 | -0.550 | 0.583 | 0.978 |
| Inferior temporal | -0.034 | 0.033 | -1.040 | 0.299 | 0.954 |  | -0.015 | 0.019 | -0.780 | 0.434 | 0.978 |
| Isthmus cingulate | 0.018 | 0.032 | 0.550 | 0.583 | 0.954 |  | 0.011 | 0.019 | 0.580 | 0.560 | 0.978 |
| Lateral occipital | 0.009 | 0.034 | 0.270 | 0.787 | 0.954 |  | -0.008 | 0.020 | -0.390 | 0.698 | 0.978 |
| Lateral orbitofrontal | 0.027 | 0.033 | 0.820 | 0.411 | 0.954 |  | 0.006 | 0.019 | 0.300 | 0.764 | 0.978 |
| Lingual | -0.028 | 0.033 | -0.860 | 0.390 | 0.954 |  | -0.005 | 0.019 | -0.270 | 0.791 | 0.978 |
| Medial orbitofrontal | -0.023 | 0.032 | -0.720 | 0.469 | 0.954 |  | 0.001 | 0.019 | 0.060 | 0.954 | 0.978 |
| Middle temporal | -0.040 | 0.032 | -1.250 | 0.212 | 0.954 |  | 0.001 | 0.019 | 0.080 | 0.939 | 0.978 |
| Parahippocampal | 0.004 | 0.032 | 0.110 | 0.913 | 0.974 |  | -0.036 | 0.019 | -1.890 | 0.058 | 0.978 |
| Paracentral | 0.005 | 0.034 | 0.140 | 0.885 | 0.974 |  | -0.007 | 0.020 | -0.340 | 0.732 | 0.978 |
| Pars opercularis | 0.057 | 0.033 | 1.750 | 0.079 | 0.954 |  | -0.012 | 0.019 | -0.630 | 0.531 | 0.978 |
| Pars orbitalis | 0.035 | 0.032 | 1.110 | 0.268 | 0.954 |  | -0.002 | 0.019 | -0.120 | 0.908 | 0.978 |
| Pars triangularis | 0.009 | 0.033 | 0.290 | 0.773 | 0.954 |  | 0.014 | 0.019 | 0.710 | 0.480 | 0.978 |
| Pericalcarine | 0.015 | 0.033 | 0.450 | 0.651 | 0.954 |  | 0.023 | 0.019 | 1.170 | 0.241 | 0.978 |
| Postcentral | 0.014 | 0.033 | 0.420 | 0.675 | 0.954 |  | 0.004 | 0.019 | 0.210 | 0.834 | 0.978 |
| Posterior cingulate | -0.014 | 0.031 | -0.460 | 0.647 | 0.954 |  | 0.013 | 0.018 | 0.730 | 0.464 | 0.978 |
| Precentral | 0.036 | 0.034 | 1.070 | 0.287 | 0.954 |  | -0.002 | 0.020 | -0.130 | 0.899 | 0.978 |
| Precuneus | 0.011 | 0.034 | 0.340 | 0.736 | 0.954 |  | 0.004 | 0.020 | 0.220 | 0.828 | 0.978 |
| Rostral anterior cingulate | 0.014 | 0.031 | 0.470 | 0.636 | 0.954 |  | -0.029 | 0.018 | -1.620 | 0.106 | 0.978 |
| Rostral middle frontal | 0.022 | 0.034 | 0.640 | 0.521 | 0.954 |  | -0.003 | 0.020 | -0.140 | 0.887 | 0.978 |
| Superior frontal | 0.011 | 0.033 | 0.320 | 0.746 | 0.954 |  | -0.012 | 0.019 | -0.640 | 0.520 | 0.978 |
| Superior parietal | 0.003 | 0.034 | 0.090 | 0.925 | 0.974 |  | -0.001 | 0.020 | -0.070 | 0.948 | 0.978 |
| Superior temporal | 0.009 | 0.033 | 0.270 | 0.785 | 0.954 |  | -0.006 | 0.019 | -0.310 | 0.753 | 0.978 |
| Supramarginal | 0.041 | 0.033 | 1.240 | 0.217 | 0.954 |  | 0.007 | 0.019 | 0.380 | 0.703 | 0.978 |
| Transverse temporal | 0.041 | 0.033 | 1.250 | 0.210 | 0.954 |  | 0.000 | 0.019 | <0.001 | 0.998 | 0.998 |
| Insula | 0.033 | 0.033 | 0.980 | 0.326 | 0.954 |  | -0.003 | 0.019 | -0.170 | 0.867 | 0.978 |
| **Fractional anisotropy** |  |  |  |  |  |  |  |  |  |  |  |
| Forceps major | 0.077 | 0.038 | 2.030 | 0.042 | 0.095 |  | -0.031 | 0.022 | -1.400 | 0.163 | 0.623 |
| Forceps minor | 0.077 | 0.037 | 2.090 | 0.036 | 0.094 |  | -0.022 | 0.021 | -1.030 | 0.301 | 0.758 |
| Middle cerebellar peduncle | 0.056 | 0.038 | 1.470 | 0.141 | 0.228 |  | -0.038 | 0.022 | -1.720 | 0.085 | 0.623 |
| Acoustic radiation | -0.003 | 0.032 | -0.100 | 0.923 | 0.951 |  | -0.040 | 0.019 | -2.080 | 0.037 | 0.555 |
| Anterior thalamic radiation | 0.042 | 0.035 | 1.200 | 0.230 | 0.326 |  | -0.023 | 0.021 | -1.130 | 0.257 | 0.758 |
| Cingulate gyrus part of cingulum | 0.032 | 0.033 | 0.980 | 0.325 | 0.409 |  | -0.022 | 0.019 | -1.140 | 0.254 | 0.758 |
| Parahippocampal part of cingulum | 0.011 | 0.032 | 0.330 | 0.744 | 0.816 |  | -0.007 | 0.019 | -0.380 | 0.705 | 0.824 |
| Corticospinal tract | 0.014 | 0.034 | 0.420 | 0.676 | 0.766 |  | -0.030 | 0.020 | -1.480 | 0.140 | 0.623 |
| Inferior fronto-occipital fasciculus | 0.095 | 0.036 | 2.660 | 0.008 | 0.054 |  | -0.030 | 0.021 | -1.440 | 0.150 | 0.623 |
| Inferior longitudinal fasciculus | 0.086 | 0.036 | 2.410 | 0.016 | 0.064 |  | -0.032 | 0.021 | -1.510 | 0.130 | 0.623 |
| Medial lemniscus | -0.007 | 0.032 | -0.210 | 0.831 | 0.883 |  | -0.019 | 0.018 | -1.030 | 0.303 | 0.758 |
| Posterior thalamic radiation | 0.057 | 0.035 | 1.640 | 0.100 | 0.189 |  | -0.049 | 0.020 | -2.420 | 0.016 | 0.480 |
| Superior longitudinal fasciculus | 0.086 | 0.036 | 2.380 | 0.017 | 0.064 |  | -0.012 | 0.021 | -0.560 | 0.576 | 0.824 |
| Superior thalamic radiation | 0.033 | 0.036 | 0.910 | 0.365 | 0.443 |  | -0.029 | 0.021 | -1.380 | 0.166 | 0.623 |
| Uncinate fasciculus | 0.069 | 0.033 | 2.110 | 0.035 | 0.094 |  | -0.017 | 0.019 | -0.910 | 0.364 | 0.758 |
| **Mean diffusivity** |  |  |  |  |  |  |  |  |  |  |  |
| Forceps major | -0.063 | 0.037 | -1.720 | 0.086 | 0.172 |  | 0.009 | 0.022 | 0.430 | 0.670 | 0.824 |
| Forceps minor | -0.103 | 0.036 | -2.900 | 0.004 | 0.043 |  | 0.008 | 0.021 | 0.370 | 0.714 | 0.824 |
| Middle cerebellar peduncle | -0.001 | 0.037 | -0.040 | 0.970 | 0.970 |  | -0.020 | 0.022 | -0.920 | 0.357 | 0.758 |
| Acoustic radiation | -0.033 | 0.032 | -1.010 | 0.311 | 0.407 |  | 0.002 | 0.019 | 0.130 | 0.896 | 0.896 |
| Acoustic radiation (left) | -0.018 | 0.038 | -0.470 | 0.640 | 0.750 |  | — | — | — | — | — |
| Acoustic radiation (right) | -0.050 | 0.038 | -1.330 | 0.185 | 0.286 |  | — | — | — | — | — |
| Anterior thalamic radiation | -0.082 | 0.033 | -2.510 | 0.012 | 0.063 |  | -0.006 | 0.019 | -0.320 | 0.747 | 0.830 |
| Cingulate gyrus part of cingulum | -0.052 | 0.034 | -1.520 | 0.128 | 0.218 |  | 0.003 | 0.020 | 0.140 | 0.891 | 0.896 |
| Parahippocampal part of cingulum | -0.037 | 0.032 | -1.150 | 0.250 | 0.340 |  | 0.010 | 0.019 | 0.550 | 0.583 | 0.824 |
| Corticospinal tract | -0.042 | 0.035 | -1.220 | 0.224 | 0.326 |  | 0.005 | 0.020 | 0.240 | 0.807 | 0.865 |
| Inferior fronto-occipital fasciculus | -0.109 | 0.034 | -3.150 | 0.002 | 0.034 |  | 0.008 | 0.020 | 0.400 | 0.690 | 0.824 |
| Inferior longitudinal fasciculus | -0.096 | 0.034 | -2.810 | 0.005 | 0.043 |  | 0.008 | 0.020 | 0.410 | 0.680 | 0.824 |
| Medial lemniscus | -0.068 | 0.032 | -2.140 | 0.032 | 0.094 |  | 0.016 | 0.019 | 0.880 | 0.379 | 0.758 |
| Posterior thalamic radiation | -0.081 | 0.033 | -2.470 | 0.013 | 0.063 |  | 0.015 | 0.019 | 0.790 | 0.429 | 0.804 |
| Superior longitudinal fasciculus | -0.108 | 0.036 | -3.030 | 0.002 | 0.034 |  | 0.013 | 0.021 | 0.600 | 0.548 | 0.824 |
| Superior thalamic radiation | -0.072 | 0.032 | -2.250 | 0.025 | 0.085 |  | 0.008 | 0.019 | 0.450 | 0.655 | 0.824 |
| Uncinate fasciculus | -0.061 | 0.032 | -1.910 | 0.057 | 0.121 |  | 0.009 | 0.019 | 0.470 | 0.639 | 0.824 |
| Uncinate fasciculus (left) | -0.053 | 0.033 | -1.620 | 0.106 | 0.190 |  | — | — | — | — | — |
| Uncinate fasciculus (right) | -0.072 | 0.035 | -2.060 | 0.040 | 0.095 |  | — | — | — | — | — |

**Table S6. The associations between anhedonia and brain structures in the Cheadle and Newcastle imaging centers.** The model was conducted with age, age^2^, sex, total brain volume and head position coordinates set as covariates. Hemisphere was also set as a covariate when appropriate.

| **Outcome** | **Cheadle** | | | |  | **Newcastle** | | | |
| --- | --- | --- | --- | --- | --- | --- | --- | --- | --- |
|  | **β** | **SE** | **Z** | **p** |  | **β** | **SE** | **Z** | **p** |
| Total grey matter volume | -0.028 | 0.005 | -5.32 | <0.001 |  | -0.015 | 0.011 | -1.33 | 0.185 |
| Total white matter volume | 0.018 | 0.005 | 3.77 | <0.001 |  | 0.013 | 0.010 | 1.34 | 0.180 |
| Thalamus | -0.044 | 0.010 | -4.60 | <0.001 |  | -0.022 | 0.020 | -1.05 | 0.294 |
| Accumbens | -0.053 | 0.012 | -4.43 | <0.001 |  | -0.033 | 0.026 | -1.26 | 0.207 |
| **Cortical thickness** |  |  |  |  |  |  |  |  |  |
| Paracentral | -0.041 | 0.014 | -2.92 | 0.004 |  | -0.037 | 0.031 | -1.18 | 0.237 |
| Pars opercularis | -0.048 | 0.014 | -3.50 | <0.001 |  | 0.004 | 0.028 | 0.14 | 0.887 |
| Precentral | -0.038 | 0.014 | -2.72 | 0.006 |  | -0.009 | 0.031 | -0.29 | 0.775 |
| Rostral anterior cingulate | -0.046 | 0.013 | -3.59 | <0.001 |  | -0.021 | 0.027 | -0.77 | 0.443 |
| Insula | -0.044 | 0.014 | -3.17 | 0.002 |  | -0.009 | 0.030 | -0.30 | 0.764 |
| **Fractional anisotropy** |  |  |  |  |  |  |  |  |  |
| Forceps major | -0.059 | 0.016 | -3.61 | <0.001 |  | 0.022 | 0.035 | 0.62 | 0.537 |
| Anterior thalamic radiation | -0.047 | 0.015 | -3.13 | 0.002 |  | >-0.001 | 0.033 | -0.01 | 0.995 |
| Inferior longitudinal fasciculus | -0.054 | 0.015 | -3.54 | <0.001 |  | 0.030 | 0.033 | 0.90 | 0.367 |
| Posterior thalamic radiation | -0.064 | 0.015 | -4.30 | <0.001 |  | -0.011 | 0.033 | -0.34 | 0.731 |
| Superior longitudinal fasciculus | -0.050 | 0.015 | -3.27 | 0.001 |  | 0.028 | 0.033 | 0.86 | 0.392 |
| **Mean diffusivity** |  |  |  |  |  |  |  |  |  |
| Forceps major | 0.059 | 0.016 | 3.73 | <0.001 |  | -0.008 | 0.034 | -0.23 | 0.815 |
| Forceps minor | 0.054 | 0.015 | 3.49 | <0.001 |  | -0.036 | 0.031 | -1.17 | 0.240 |
| Acoustic radiation (left) | 0.040 | 0.017 | 2.40 | 0.016 |  | 0.017 | 0.031 | 0.54 | 0.592 |
| Anterior thalamic radiation | 0.080 | 0.014 | 5.64 | <0.001 |  | -0.002 | 0.029 | -0.06 | 0.951 |
| Cingulate gyrus part of cingulum | 0.061 | 0.015 | 4.11 | <0.001 |  | 0.014 | 0.030 | 0.48 | 0.631 |
| Corticospinal tract | 0.059 | 0.015 | 3.89 | <0.001 |  | 0.014 | 0.031 | 0.45 | 0.651 |
| Inferior fronto-occipital fasciculus | 0.069 | 0.015 | 4.62 | <0.001 |  | -0.035 | 0.031 | -1.12 | 0.265 |
| Inferior longitudinal fasciculus | 0.058 | 0.014 | 4.12 | <0.001 |  | -0.011 | 0.030 | -0.36 | 0.718 |
| Posterior thalamic radiation | 0.082 | 0.015 | 5.32 | <0.001 |  | -0.021 | 0.032 | -0.64 | 0.524 |
| Superior longitudinal fasciculus | 0.079 | 0.014 | 5.66 | <0.001 |  | 0.012 | 0.029 | 0.41 | 0.683 |
| Superior thalamic radiation | 0.056 | 0.014 | 4.02 | <0.001 |  | -0.010 | 0.029 | -0.36 | 0.720 |
| Uncinate fasciculus | 0.040 | 0.014 | 2.84 | 0.004 |  | -0.018 | 0.030 | -0.62 | 0.534 |
| Uncinate fasciculus (left) | 0.072 | 0.015 | 4.71 | <0.001 |  | -0.005 | 0.031 | -0.17 | 0.864 |
| Uncinate fasciculus (right) | -0.028 | 0.005 | -5.32 | <0.001 |  | -0.015 | 0.011 | -1.33 | 0.185 |

**Table S7. The associations between PRS-anhedonia and brain structures in the Cheadle and Newcastle imaging centers.** The model was conducted with age, age^2^, sex, total brain volume, head position coordinates, genotype array, the first ten genetic principal components and assessment center set as covariates. Hemisphere was also set as a covariate when appropriate.

| **Outcome** | **Cheadle** | | | |  | **Newcastle** | | | |
| --- | --- | --- | --- | --- | --- | --- | --- | --- | --- |
|  | **β** | **SE** | **Z** | **p** |  | **β** | **SE** | **Z** | **p** |
| Total grey matter volume | -0.014 | 0.003 | -4.93 | <0.001 |  | -0.017 | 0.007 | -2.44 | 0.015 |
| Total white matter volume | 0.012 | 0.003 | 4.75 | <0.001 |  | 0.015 | 0.006 | 2.48 | 0.013 |
| **Cortical thickness** |  |  |  |  |  |  |  |  |  |
| Parahippocampal | -0.026 | 0.007 | -3.52 | <0.001 |  | -0.061 | 0.017 | -3.56 | <0.001 |
| Superior temporal | -0.023 | 0.007 | -3.17 | 0.002 |  | -0.031 | 0.017 | -1.86 | 0.063 |
| Insula | -0.029 | 0.007 | -3.83 | <0.001 |  | -0.034 | 0.018 | -1.95 | 0.051 |
| **Fractional anisotropy** |  |  |  |  |  |  |  |  |  |
| Forceps minor | -0.019 | 0.009 | -2.26 | 0.024 |  | -0.038 | 0.020 | -1.94 | 0.053 |
| Posterior thalamic radiation | -0.010 | 0.008 | -1.28 | 0.199 |  | -0.061 | 0.019 | -3.14 | 0.002 |
| **Mean diffusivity** |  |  |  |  |  |  |  |  |  |
| Forceps minor | 0.021 | 0.008 | 2.51 | 0.012 |  | 0.026 | 0.018 | 1.45 | 0.148 |
| Middle cerebellar peduncle | 0.026 | 0.009 | 3.00 | 0.003 |  | 0.008 | 0.021 | 0.38 | 0.705 |
| Anterior thalamic radiation | 0.021 | 0.008 | 2.73 | 0.006 |  | 0.015 | 0.017 | 0.87 | 0.385 |
| Cingulate gyrus part of cingulum | 0.019 | 0.008 | 2.34 | 0.019 |  | 0.022 | 0.017 | 1.27 | 0.202 |
| Inferior fronto-occipital fasciculus | 0.018 | 0.008 | 2.25 | 0.024 |  | 0.027 | 0.018 | 1.45 | 0.146 |
| Inferior longitudinal fasciculus | 0.015 | 0.008 | 1.93 | 0.053 |  | 0.025 | 0.018 | 1.36 | 0.175 |
| Posterior thalamic radiation | 0.022 | 0.008 | 2.84 | 0.004 |  | 0.038 | 0.018 | 2.16 | 0.031 |
| Superior longitudinal fasciculus | 0.017 | 0.008 | 2.08 | 0.038 |  | 0.031 | 0.019 | 1.62 | 0.105 |
| Superior thalamic radiation | 0.015 | 0.008 | 2.01 | 0.045 |  | 0.025 | 0.017 | 1.45 | 0.148 |
| Uncinate fasciculus | 0.018 | 0.007 | 2.42 | 0.015 |  | 0.026 | 0.017 | 1.54 | 0.123 |

Table S8. The association between anhedonia as a dichotomous variable and significant regions from the main analysis of anhedonia. The same model was conducted with age, age^2^, sex, total ICV and head position coordinates set as covariates. Hemisphere was also set as a covariate when appropriate.

| **Outcome** | **β** | **SE** | **Z** | **P** | **p_corrected_** |
| --- | --- | --- | --- | --- | --- |
| grey matter | -0.033 | 0.006 | -5.140 | <0.001 | <0.001 |
| white matter | 0.020 | 0.006 | 3.430 | 0.001 | 0.002 |
| Thalamus | -0.056 | 0.012 | -4.690 | <0.001 | <0.001 |
| Accumbens | -0.069 | 0.015 | -4.600 | <0.001 | <0.001 |
| Paracentral | -0.035 | 0.018 | -1.990 | 0.047 | 0.054 |
| Pars opercularis | -0.048 | 0.017 | -2.820 | 0.005 | 0.009 |
| Precentral | -0.024 | 0.018 | -1.380 | 0.167 | 0.167 |
| Rostral anterior cingulate | -0.043 | 0.016 | -2.660 | 0.008 | 0.012 |
| Insula | -0.034 | 0.017 | -1.970 | 0.048 | 0.054 |
| Forceps major | -0.045 | 0.020 | -2.250 | 0.025 | 0.028 |
| Anterior thalamic radiation | -0.043 | 0.019 | -2.300 | 0.021 | 0.025 |
| Inferior longitudinal fasciculus | -0.046 | 0.019 | -2.400 | 0.017 | 0.022 |
| Posterior thalamic radiation | -0.074 | 0.019 | -3.960 | <0.001 | <0.001 |
| Superior longitudinal fasciculus | -0.049 | 0.019 | -2.560 | 0.011 | 0.017 |
| Forceps major | 0.049 | 0.020 | 2.490 | 0.013 | 0.019 |
| Forceps minor | 0.047 | 0.019 | 2.440 | 0.015 | 0.020 |
| Acoustic radiation (left) | 0.037 | 0.020 | 1.830 | 0.068 | 0.072 |
| Anterior thalamic radiation | 0.080 | 0.017 | 4.580 | <0.001 | <0.001 |
| Cingulate gyrus part of cingulum | 0.054 | 0.018 | 2.990 | 0.003 | 0.006 |
| Corticospinal tract | 0.057 | 0.019 | 3.030 | 0.002 | 0.005 |
| Inferior fronto-occipital fasciculus | 0.059 | 0.018 | 3.200 | 0.001 | 0.003 |
| Inferior longitudinal fasciculus | 0.053 | 0.018 | 2.910 | 0.004 | 0.008 |
| Posterior thalamic radiation | 0.059 | 0.018 | 3.320 | 0.001 | 0.003 |
| Superior longitudinal fasciculus | 0.085 | 0.019 | 4.470 | <0.001 | <0.001 |
| Superior thalamic radiation | 0.088 | 0.017 | 5.100 | <0.001 | <0.001 |
| Uncinate fasciculus | 0.046 | 0.017 | 2.680 | 0.007 | 0.012 |
| Uncinate fasciculus (left) | 0.029 | 0.018 | 1.620 | 0.104 | 0.104 |
| Uncinate fasciculus (right) | 0.063 | 0.019 | 3.360 | 0.001 | 0.003 |

Table S9. The association between anhedonia and significant regions emerged from the main analysis of anhedonia in healthy individuals. The same model was conducted with age, age^2^, sex, total ICV and head position coordinates set as covariates. Hemisphere was also set as a covariate when appropriate.

| **Outcome** | **N** | **β** | **SE** | **Z-value** | **p** | **p_corrected_** |
| --- | --- | --- | --- | --- | --- | --- |
| grey matter | 17 464 | -0.018 | 0.006 | -3.220 | 0.001 | 0.002 |
| white matter | 17 452 | 0.011 | 0.005 | 2.170 | 0.030 | 0.034 |
| Thalamus | 17 437 | -0.041 | 0.010 | -4.000 | <0.001 | <0.001 |
| Accumbens | 17 473 | -0.057 | 0.013 | -4.470 | <0.001 | <0.001 |
| Paracentral | 17 249 | -0.044 | 0.015 | -2.920 | 0.003 | 0.005 |
| Pars opercularis | 17 200 | -0.040 | 0.015 | -2.760 | 0.006 | 0.009 |
| Precentral | 17 224 | -0.032 | 0.015 | -2.110 | 0.035 | 0.035 |
| Rostral anterior cingulate | 17 260 | -0.045 | 0.014 | -3.290 | 0.001 | 0.002 |
| Insula | 17 250 | -0.033 | 0.015 | -2.230 | 0.026 | 0.033 |
| Forceps major | 15 698 | -0.036 | 0.017 | -2.070 | 0.038 | 0.103 |
| Anterior thalamic radiation | 15 793 | -0.018 | 0.016 | -1.130 | 0.260 | 0.309 |
| Inferior longitudinal fasciculus | 15 801 | -0.022 | 0.016 | -1.330 | 0.185 | 0.284 |
| Posterior thalamic radiation | 15 802 | -0.045 | 0.016 | -2.830 | 0.005 | 0.048 |
| Superior longitudinal fasciculus | 15 800 | -0.016 | 0.016 | -1.000 | 0.317 | 0.335 |
| Forceps major | 15 675 | 0.030 | 0.017 | 1.790 | 0.073 | 0.173 |
| Forceps minor | 15 725 | 0.019 | 0.016 | 1.170 | 0.242 | 0.308 |
| Acoustic radiation (left) | 15 703 | 0.020 | 0.017 | 1.170 | 0.243 | 0.308 |
| Anterior thalamic radiation | 15 659 | 0.034 | 0.015 | 2.250 | 0.024 | 0.089 |
| Cingulate gyrus part of cingulum | 15 759 | 0.021 | 0.016 | 1.330 | 0.183 | 0.284 |
| Corticospinal tract | 15 798 | 0.039 | 0.016 | 2.440 | 0.015 | 0.089 |
| Inferior fronto-occipital fasciculus | 15 766 | 0.024 | 0.016 | 1.510 | 0.132 | 0.251 |
| Inferior longitudinal fasciculus | 15 749 | 0.020 | 0.016 | 1.300 | 0.194 | 0.284 |
| Posterior thalamic radiation | 15 687 | 0.033 | 0.015 | 2.200 | 0.028 | 0.089 |
| Superior longitudinal fasciculus | 15 699 | 0.037 | 0.016 | 2.270 | 0.023 | 0.089 |
| Superior thalamic radiation | 15 639 | 0.043 | 0.015 | 2.910 | 0.004 | 0.048 |
| Uncinate fasciculus | 15 807 | 0.015 | 0.015 | 1.010 | 0.311 | 0.335 |
| Uncinate fasciculus (left) | 15 729 | 0.002 | 0.015 | 0.150 | 0.878 | 0.878 |
| Uncinate fasciculus (right) | 15 714 | 0.026 | 0.016 | 1.640 | 0.100 | 0.211 |

Table S10. The interaction between anhedonia and mental health status on significant measures from the main analysis for anhedonia. Age, age^2^, sex, total ICV and lateral, transverse and longitudinal scanner position coordinates were set as covariates. Anhedonia, mental health status and the interaction between anhedonia and mental health status were also included in the model. Hemisphere was also set as a covariate when appropriate.

| **Outcome** | **β** | **SE** | **Z-value** | **p** | **p_corrected_** |
| --- | --- | --- | --- | --- | --- |
| grey matter | -0.025 | 0.011 | -2.260 | 0.024 | 0.108 |
| white matter | 0.025 | 0.010 | 2.530 | 0.012 | 0.108 |
| Thalamus | 0.009 | 0.021 | 0.430 | 0.666 | 0.856 |
| Accumbens | 0.032 | 0.026 | 1.240 | 0.214 | 0.642 |
| Paracentral | 0.008 | 0.030 | 0.270 | 0.784 | 0.882 |
| Pars opercularis | 0.003 | 0.029 | 0.120 | 0.908 | 0.908 |
| Precentral | -0.016 | 0.030 | -0.530 | 0.599 | 0.856 |
| Rostral anterior cingulate | 0.016 | 0.027 | 0.590 | 0.557 | 0.856 |
| Insula | -0.017 | 0.030 | -0.580 | 0.561 | 0.856 |
| Forceps major | 0.005 | 0.035 | 0.140 | 0.890 | 0.890 |
| Anterior thalamic radiation | -0.042 | 0.032 | -1.310 | 0.192 | 0.304 |
| Inferior longitudinal fasciculus | -0.046 | 0.033 | -1.390 | 0.164 | 0.283 |
| Posterior thalamic radiation | -0.015 | 0.032 | -0.460 | 0.642 | 0.681 |
| Superior longitudinal fasciculus | -0.072 | 0.033 | -2.180 | 0.029 | 0.069 |
| Forceps major | 0.035 | 0.034 | 1.030 | 0.305 | 0.446 |
| Forceps minor | 0.032 | 0.033 | 0.980 | 0.329 | 0.447 |
| Acoustic radiation (left) | 0.029 | 0.035 | 0.840 | 0.401 | 0.508 |
| Anterior thalamic radiation | 0.061 | 0.030 | 2.050 | 0.041 | 0.087 |
| Cingulate gyrus part of cingulum | 0.078 | 0.031 | 2.510 | 0.012 | 0.065 |
| Corticospinal tract | 0.015 | 0.032 | 0.460 | 0.645 | 0.681 |
| Inferior fronto-occipital fasciculus | 0.070 | 0.032 | 2.220 | 0.026 | 0.069 |
| Inferior longitudinal fasciculus | 0.076 | 0.031 | 2.440 | 0.015 | 0.065 |
| Posterior thalamic radiation | 0.022 | 0.030 | 0.710 | 0.478 | 0.568 |
| Superior longitudinal fasciculus | 0.078 | 0.033 | 2.400 | 0.017 | 0.065 |
| Superior thalamic radiation | 0.058 | 0.029 | 1.970 | 0.049 | 0.093 |
| Uncinate fasciculus | 0.071 | 0.029 | 2.430 | 0.015 | 0.065 |
| Uncinate fasciculus (left) | 0.067 | 0.030 | 2.210 | 0.027 | 0.069 |
| Uncinate fasciculus (right) | 0.080 | 0.032 | 2.490 | 0.013 | 0.065 |

Table S11. The association between anhedonia and significant regions emerged from the main analysis of anhedonia, with age, age^2^, sex, total brain volume head position coordinates, anhedonia, depressed mood, childhood traumatic events, adulthood traumatic events, medication use, body mass index, current tobacco use, alcohol intake frequency, education qualification and Townsend deprivation index considered as covariates. Hemisphere was also set as a covariate when appropriate.

| **Outcome** | **N** | **β** | **SE** | **Z-value** | **p** | **p_corrected_** |
| --- | --- | --- | --- | --- | --- | --- |
| grey matter | 12,472 | -0.025 | 0.008 | -3.070 | 0.002 | 0.009 |
| white matter | 12,466 | 0.018 | 0.007 | 2.520 | 0.012 | 0.022 |
| Thalamus | 12,458 | -0.015 | 0.015 | -0.970 | 0.333 | 0.333 |
| Accumbens | 12,479 | -0.043 | 0.019 | -2.240 | 0.025 | 0.038 |
| Paracentral | 12,322 | -0.062 | 0.022 | -2.810 | 0.005 | 0.011 |
| Pars opercularis | 12,291 | -0.070 | 0.022 | -3.220 | 0.001 | 0.009 |
| Precentral | 12,309 | -0.063 | 0.022 | -2.830 | 0.005 | 0.011 |
| Rostral anterior cingulate | 12,329 | -0.029 | 0.020 | -1.430 | 0.153 | 0.172 |
| Insula | 12,327 | -0.041 | 0.022 | -1.870 | 0.061 | 0.078 |
| Forceps major | 11,250 | -0.001 | 0.026 | -0.040 | 0.970 | 0.994 |
| Anterior thalamic radiation | 11,310 | -0.015 | 0.024 | -0.610 | 0.544 | 0.820 |
| Inferior longitudinal fasciculus | 11,327 | -0.012 | 0.024 | -0.480 | 0.629 | 0.820 |
| Posterior thalamic radiation | 11,322 | 0.000 | 0.024 | -0.010 | 0.994 | 0.994 |
| Superior longitudinal fasciculus | 11,317 | -0.028 | 0.024 | -1.150 | 0.251 | 0.667 |
| Forceps major | 11,218 | 0.034 | 0.025 | 1.340 | 0.180 | 0.667 |
| Forceps minor | 11,274 | 0.017 | 0.024 | 0.710 | 0.478 | 0.820 |
| Acoustic radiation (left) | 11,239 | 0.014 | 0.026 | 0.530 | 0.596 | 0.820 |
| Anterior thalamic radiation | 11,230 | 0.024 | 0.022 | 1.080 | 0.281 | 0.667 |
| Cingulate gyrus part of cingulum | 11,298 | 0.016 | 0.023 | 0.690 | 0.488 | 0.820 |
| Corticospinal tract | 11,311 | 0.006 | 0.024 | 0.270 | 0.786 | 0.895 |
| Inferior fronto-occipital fasciculus | 11,299 | 0.006 | 0.023 | 0.250 | 0.801 | 0.895 |
| Inferior longitudinal fasciculus | 11,282 | 0.014 | 0.023 | 0.600 | 0.546 | 0.820 |
| Posterior thalamic radiation | 11,256 | 0.010 | 0.022 | 0.460 | 0.647 | 0.820 |
| Superior longitudinal fasciculus | 11,259 | 0.028 | 0.024 | 1.170 | 0.243 | 0.667 |
| Superior thalamic radiation | 11,213 | 0.039 | 0.022 | 1.810 | 0.071 | 0.519 |
| Uncinate fasciculus | 11,320 | 0.038 | 0.022 | 1.740 | 0.082 | 0.519 |
| Uncinate fasciculus (left) | 11,272 | 0.033 | 0.023 | 1.460 | 0.146 | 0.667 |
| Uncinate fasciculus (right) | 11,257 | 0.047 | 0.024 | 1.980 | 0.048 | 0.519 |

Table S12. The associations between PRS-anhedonia and brain structure controlling for extra confounding factors. The models were conducted with age, age^2^, sex, total brain volume head position coordinates, anhedonia, depressed mood, childhood traumatic events, adulthood traumatic events, medication use, body mass index, current tobacco use, alcohol intake frequency, education qualification and Townsend deprivation index, genotype array and the first ten genetic principal components as covariates. Hemisphere was also set as a covariate when appropriate.

| **Outcome** | **N** | **β** | **SE** | **Z-value** | **p** | **p_corrected_** |
| --- | --- | --- | --- | --- | --- | --- |
| grey matter | 10 640 | -0.007 | 0.003 | -2.330 | 0.020 | 0.020 |
| white matter | 10 633 | 0.009 | 0.003 | 3.000 | 0.003 | 0.005 |
| Parahippocampal | 10 512 | -0.031 | 0.009 | -3.540 | <0.001 | <0.001 |
| Superior temporal | 10 503 | -0.023 | 0.009 | -2.630 | 0.009 | 0.011 |
| Insula | 10 510 | -0.027 | 0.009 | -3.080 | 0.002 | 0.005 |
| Forceps minor | 9 644 | -0.020 | 0.010 | -1.980 | 0.048 | 0.057 |
| Posterior thalamic radiation | 9 661 | -0.009 | 0.009 | -0.980 | 0.326 | 0.326 |
| Forceps minor | 9 620 | 0.019 | 0.010 | 1.950 | 0.052 | 0.057 |
| Middle cerebellar peduncle | 9 576 | 0.024 | 0.010 | 2.430 | 0.015 | 0.057 |
| Anterior thalamic radiation | 9 577 | 0.020 | 0.009 | 2.250 | 0.025 | 0.057 |
| Cingulate gyrus part of cingulum | 9 638 | 0.026 | 0.009 | 2.780 | 0.005 | 0.057 |
| Inferior fronto-occipital fasciculus | 9 638 | 0.021 | 0.009 | 2.210 | 0.027 | 0.057 |
| Inferior longitudinal fasciculus | 9 620 | 0.018 | 0.009 | 2.010 | 0.044 | 0.057 |
| Posterior thalamic radiation | 9 598 | 0.019 | 0.009 | 2.190 | 0.029 | 0.057 |
| Superior longitudinal fasciculus | 9 601 | 0.021 | 0.010 | 2.160 | 0.030 | 0.057 |
| Superior thalamic radiation | 9 563 | 0.018 | 0.009 | 2.120 | 0.034 | 0.057 |
| Uncinate fasciculus | 9 655 | 0.018 | 0.009 | 2.020 | 0.044 | 0.057 |

Table S13. The associations between PRS-MDD and subcortical volumes, whole-surface cortical thickness and white matter integrity. The models of anhedonia were conducted with age, age^2^, sex, total brain volume, head position coordinates, genotype array and the first ten genetic principal components set as covariates. Hemisphere was also set as a covariate when appropriate.

| **Outcome** | **N** | **β** | **SE** | **Z** | **p** | **p_corrected_** |
| --- | --- | --- | --- | --- | --- | --- |
| grey matter | 16617 | -0.002 | 0.003 | -0.920 | 0.356 | 0.843 |
| white matter | 16604 | 0.002 | 0.002 | 0.760 | 0.445 | 0.843 |
| Thalamus | 16566 | -0.007 | 0.005 | -1.440 | 0.149 | 0.662 |
| Caudate | 16536 | 0.007 | 0.006 | 1.090 | 0.277 | 0.843 |
| Putamen | 16548 | 0.007 | 0.006 | 1.260 | 0.206 | 0.687 |
| Pallidum | 16433 | -0.009 | 0.006 | -1.510 | 0.132 | 0.662 |
| Hippocampus | 16454 | <0.001 | 0.006 | 0.020 | 0.983 | 0.983 |
| Amygdala | 16555 | -0.006 | 0.006 | -0.970 | 0.332 | 0.843 |
| Accumbens | 16558 | -0.001 | 0.006 | -0.100 | 0.919 | 0.967 |
| **Cortical thickness** | |  |  |  |  |  |
| Caudal anterior cingulate | 16276 | -0.003 | 0.006 | -0.530 | 0.598 | 0.886 |
| Caudal middle frontal | 16284 | 0.004 | 0.007 | 0.600 | 0.548 | 0.843 |
| Cuneus | 16366 | 0.009 | 0.007 | 1.300 | 0.194 | 0.687 |
| Entorhinal | 16218 | 0.005 | 0.007 | 0.700 | 0.482 | 0.843 |
| Fusiform | 16323 | 0.002 | 0.007 | 0.260 | 0.798 | 0.960 |
| Inferior parietal | 16241 | 0.013 | 0.007 | 1.840 | 0.065 | 0.608 |
| Inferior temporal | 16321 | -0.003 | 0.007 | -0.390 | 0.695 | 0.956 |
| Isthmus cingulate | 16338 | -0.004 | 0.007 | -0.610 | 0.545 | 0.843 |
| Lateral occipital | 16353 | 0.017 | 0.007 | 2.370 | 0.018 | 0.360 |
| Lateral orbitofrontal | 16348 | -0.005 | 0.007 | -0.760 | 0.445 | 0.843 |
| Lingual | 16364 | 0.012 | 0.007 | 1.770 | 0.076 | 0.608 |
| Medial orbitofrontal | 16346 | -0.005 | 0.007 | -0.760 | 0.450 | 0.843 |
| Middle temporal | 16360 | 0.002 | 0.007 | 0.350 | 0.730 | 0.956 |
| Parahippocampal | 16359 | -0.002 | 0.007 | -0.290 | 0.770 | 0.960 |
| Paracentral | 16339 | 0.002 | 0.007 | 0.330 | 0.741 | 0.956 |
| Pars opercularis | 16263 | <0.001 | 0.007 | -0.060 | 0.953 | 0.977 |
| Pars orbitalis | 16310 | -0.006 | 0.007 | -0.920 | 0.359 | 0.843 |
| Pars triangularis | 16232 | -0.001 | 0.007 | -0.150 | 0.878 | 0.967 |
| Pericalcarine | 16369 | 0.010 | 0.007 | 1.470 | 0.142 | 0.662 |
| Postcentral | 16345 | 0.010 | 0.007 | 1.480 | 0.139 | 0.662 |
| Posterior cingulate | 16358 | -0.016 | 0.007 | -2.480 | 0.013 | 0.360 |
| Precentral | 16284 | 0.002 | 0.007 | 0.230 | 0.816 | 0.960 |
| Precuneus | 16313 | 0.007 | 0.007 | 0.920 | 0.356 | 0.843 |
| Rostral anterior cingulate | 16328 | -0.004 | 0.006 | -0.640 | 0.521 | 0.843 |
| Rostral middle frontal | 16211 | 0.001 | 0.007 | 0.120 | 0.902 | 0.967 |
| Superior frontal | 16284 | 0.001 | 0.007 | 0.110 | 0.914 | 0.967 |
| Superior parietal | 16284 | 0.010 | 0.007 | 1.390 | 0.166 | 0.664 |
| Superior temporal | 16340 | -0.006 | 0.007 | -0.840 | 0.399 | 0.843 |
| Supramarginal | 16261 | 0.005 | 0.007 | 0.670 | 0.501 | 0.843 |
| Transverse temporal | 16383 | 0.003 | 0.007 | 0.490 | 0.624 | 0.891 |
| Insula | 16343 | -0.015 | 0.007 | -2.100 | 0.036 | 0.480 |
| **Fractional anisotropy** | |  |  |  |  |  |
| Forceps major | 14938 | -0.001 | 0.008 | -0.150 | 0.883 | 0.913 |
| Forceps minor | 15001 | -0.016 | 0.008 | -2.050 | 0.040 | 0.092 |
| Middle cerebellar peduncle | 14901 | -0.003 | 0.008 | -0.310 | 0.755 | 0.809 |
| Acoustic radiation | 15004 | 0.006 | 0.007 | 0.880 | 0.380 | 0.493 |
| Anterior thalamic radiation | 14980 | -0.008 | 0.008 | -1.110 | 0.267 | 0.381 |
| Cingulate gyrus part of cingulum | 15033 | -0.008 | 0.007 | -1.180 | 0.239 | 0.377 |
| Parahippocampal part of cingulum | 14694 | 0.004 | 0.007 | 0.550 | 0.584 | 0.649 |
| Corticospinal tract | 14998 | -0.008 | 0.007 | -1.010 | 0.311 | 0.424 |
| Inferior fronto-occipital fasciculus | 15003 | -0.010 | 0.008 | -1.300 | 0.194 | 0.342 |
| Inferior longitudinal fasciculus | 14983 | -0.012 | 0.008 | -1.580 | 0.115 | 0.230 |
| Medial lemniscus | 14983 | -0.005 | 0.007 | -0.800 | 0.427 | 0.493 |
| Posterior thalamic radiation | 14962 | -0.019 | 0.008 | -2.540 | 0.011 | 0.036 |
| Superior longitudinal fasciculus | 14990 | -0.009 | 0.008 | -1.220 | 0.224 | 0.373 |
| Superior thalamic radiation | 14979 | -0.014 | 0.008 | -1.810 | 0.071 | 0.152 |
| Uncinate fasciculus | 15013 | <0.001 | 0.007 | -0.010 | 0.989 | 0.989 |
| **Mean diffusivity** | |  |  |  |  |  |
| Forceps major | 14909 | 0.006 | 0.008 | 0.800 | 0.424 | 0.493 |
| Forceps minor | 14958 | 0.023 | 0.008 | 2.960 | 0.003 | 0.013 |
| Middle cerebellar peduncle | 14929 | 0.009 | 0.008 | 1.110 | 0.265 | 0.381 |
| Acoustic radiation | 14931 | 0.010 | 0.007 | 1.430 | 0.151 | 0.283 |
| Anterior thalamic radiation | 14795 | 0.018 | 0.007 | 2.520 | 0.012 | 0.036 |
| Cingulate gyrus part of cingulum | 14931 | 0.023 | 0.007 | 3.080 | 0.002 | 0.012 |
| Parahippocampal part of cingulum | 14549 | -0.015 | 0.007 | -2.090 | 0.036 | 0.090 |
| Corticospinal tract | 14959 | 0.021 | 0.008 | 2.770 | 0.006 | 0.023 |
| Inferior fronto-occipital fasciculus | 14930 | 0.022 | 0.007 | 3.030 | 0.002 | 0.012 |
| Inferior longitudinal fasciculus | 14909 | 0.022 | 0.007 | 3.000 | 0.003 | 0.013 |
| Medial lemniscus | 14970 | 0.006 | 0.007 | 0.810 | 0.415 | 0.493 |
| Posterior thalamic radiation | 14740 | 0.023 | 0.007 | 3.270 | 0.001 | 0.010 |
| Superior longitudinal fasciculus | 14848 | 0.027 | 0.008 | 3.540 | <0.001 | <0.001 |
| Superior thalamic radiation | 14796 | 0.024 | 0.007 | 3.420 | 0.001 | 0.010 |
| Uncinate fasciculus | 14959 | 0.016 | 0.007 | 2.260 | 0.024 | 0.065 |

# Supplementary References

1. Fischl, B. et al. Whole brain segmentation: automated labeling of neuroanatomical structures in the human brain. *Neuron* **33**, 341-355 (2002).

2. Fischl, B. FreeSurfer. *Neuroimage* **62**, 774-781 (2012).

3. Mikhael, S. S. & Pernet, C. A controlled comparison of thickness, volume and surface areas from multiple cortical parcellation packages. *BMC Bioinformatics* **20**, 55 (2019).

4. Jbabdi, S., Sotiropoulos, S. N., Savio, A. M., Graña, M. & Behrens, T. E. Model‐based analysis of multishell diffusion MR data for tractography: how to get over fitting problems. *Magn. Reson. Med.* **68**, 1846-1855 (2012).

5. de Groot, M. et al. Improving alignment in tract-based spatial statistics: evaluation and optimization of image registration. *Neuroimage* **76**, 400-411 (2013).

6. Vilhjálmsson, B. J. et al. Modeling linkage disequilibrium increases accuracy of polygenic risk scores. *Am. J. Hum. Genet.* **97**, 576-592 (2015).

7. Wray, N. R. et al. Genome-wide association analyses identify 44 risk variants and refine the genetic architecture of major depression. *Nature genetics* **50**, 668-681 (2018).

8. Shen, X. et al. A phenome-wide association and Mendelian Randomisation study of polygenic risk for depression in UK Biobank. *Nat. Commun.* **11**, 2301 (2020).

9. Alloza, C. et al. Psychotic-like experiences, polygenic risk scores for schizophrenia, and structural properties of the salience, default mode, and central-executive networks in healthy participants from UK Biobank. *Transl. Psychiatry* **10**, 1-13 (2020).

10. Klein, A. & Tourville, J. 101 labeled brain images and a consistent human cortical labeling protocol. *Front. Neurosci.* **6**, 171 (2012).
